# Diversity and Functional Properties of Lactic Acid Bacteria Isolated From Wild Fruits and Flowers Present in Northern Argentina

**DOI:** 10.3389/fmicb.2019.01091

**Published:** 2019-05-21

**Authors:** Luciana G. Ruiz Rodríguez, Florencia Mohamed, Juliana Bleckwedel, Roxana Medina, Luc De Vuyst, Elvira M. Hebert, Fernanda Mozzi

**Affiliations:** ^1^Technology and Development Laboratory, Centro de Referencia para Lactobacilos (CERELA)-CONICET, San Miguel de Tucumán, Tucumán, Argentina; ^2^Research Group of Industrial Microbiology and Food Biotechnology, Department of Bioengineering Sciences, Vrije Universiteit Brussel, Brussels, Belgium

**Keywords:** lactic acid bacteria, fructophilic lactic acid bacteria, *Fructobacillus*, tropical fruits, microbial diversity, functional properties, mannitol, esterases

## Abstract

Lactic acid bacteria (LAB) are capable of converting carbohydrate substrates into organic acids (mainly lactic acid) and producing a wide range of metabolites. Due to their interesting beneficial properties, LAB are widely used as starter cultures, as probiotics, and as microbial cell factories. Exploring LAB present in unknown niches may lead to the isolation of unique species or strains with relevant technological properties. Autochthonous rather than allochthonous starter cultures are preferred in the current industry of fermented food products, due to better adaptation and performance of autochthonous strains to the matrix they originate from. In this work, the lactic microbiota of eight different wild tropical types of fruits and four types of flowers were studied. The ability of the isolated strains to produce metabolites of interest to the food industry was evaluated. The presence of 21 species belonging to the genera *Enterococcus, Fructobacillus, Lactobacillus, Lactococcus, Leuconostoc*, and *Weissella* was evidenced by using culture-dependent techniques. The isolated LAB corresponded to 95 genotypically differentiated strains by applying rep-PCR and sequencing of the 16S rRNA gene; subsequently, representative strains of the different isolated species were studied for technological properties, such as fast growth rate and acidifying capacity; pectinolytic and cinnamoyl esterase activities, and absence of biogenic amine biosynthesis. Additionally, the strains' capacity to produce ethyl esters as well as mannitol was evaluated. The isolated fruit- and flower-origin LAB displayed functional properties that validate their potential use in the manufacture of fermented fruit-based products setting the background for the design of novel functional foods.

## Introduction

Lactic acid bacteria (LAB) constitute an ubiquitous bacterial group that is widespread in nature in niches of dairy (fermented), meat and vegetable origin, the gastrointestinal and urogenital tracts of humans and animals, and soil and water (Liu et al., 2014). These microorganisms are well known for their ability to produce lactic acid as the main end-product of their anaerobic metabolism and for synthesizing a wide range of metabolites that beneficially affect the nutritional, sensorial, and technological properties of fermented food products. For these reasons, LAB have been extensively used (i) as starter cultures; (ii) as probiotics; and (iii) in the production of interesting compounds (i.e., nutraceuticals), due to their versatile metabolism (Naeem et al., 2012; Emerenini et al., 2013; Ruiz Rodríguez et al., 2017a).

Studies on the microbial diversity of unexplored niches and environments have led to the isolation of an endless number of novel bacterial species, which may display special or unique technological and/or health-promoting properties (Di Cagno et al., 2009, 2013; Endo and Salminen, 2013; Olofsson et al., 2014). Among the sparsely explored sources of LAB, flowers, fruits, and raw vegetables constitute a remarkable niche, due to their daily contact with man. These raw materials possess high carbohydrate but low protein contents and a slightly acidic pH, providing a suitable niche to several microorganisms (Naeem et al., 2012). However, the microbial composition in these environments is fluctuating and depends on intrinsic (physical and nutritional conditions) and extrinsic (environmental and harvesting conditions) parameters of the plant matrix (Naeem et al., 2012; Di Cagno et al., 2013; Garcia et al., 2016).

In general, the microbial population of vegetables and fruits is between 10^5^ and 10^7^ CFU/g_;_ among which yeasts are the dominant group (10^2^-10^6^ CFU/g); LAB represent only a small part of the microbiota, ranging between 10^2^ and 10^4^ CFU/g (Di Cagno et al., 2013). To date, the LAB diversity present on fruits and flowers has been scarcely studied (Bae et al., 2006; Chambel et al., 2006; Nyanga et al., 2007; Yanagida et al., 2008; Chen et al., 2010; Neveling et al., 2012). Species belonging to the genera *Weissella, Lactobacillus, Lactococcus, Leuconostoc, Fructobacillus, Enterococcus, Pediococcus*, and *Streptococcus* have been found, among which *W. cibaria, W. confusa, Lb. brevis, Lb. plantarum, Lb. rossiae, Leuc. mesenteroides, Leuc. pseudomesenteroides, Lc. lactis, Ec. faecalis*, and *Ec. durans* have been reported as the most frequent species (Endo et al., 2009; Di Cagno et al., 2010; Askari et al., 2012; Naeem et al., 2012; Ong et al., 2012; Emerenini et al., 2013; Leong et al., 2014). Finally, fructophilic LAB (FLAB) species, such as *Lb. kunkeei, Lb. florum, F. fructosus, F. ficulneus, F. pseudoficulneus, F. durionis* and *F. tropaeoli*, have been detected in fruits, flowers, and vegetables (Edwards et al., 1998; Endo and Okada, 2008; Endo et al., 2009, 2010, 2011b, 2018). The origin of flower and fruit associated-microbiota remains still uncertain. It has been claimed that the microorganisms found in these niches may come from the environment, from pollinators visiting fruits and flowers when both coinciding in the plant at the same time, from birds fed with fruits, or from insects. Although the bacterial community present in the nectar of flowers may be affected by the atmosphere or by animals as dispersion vectors, the environmental and geographical factors, which shape microbial communities in the nectar, are still unknown (Alvarez-Perez et al., 2012; Fridman et al., 2012; Samuni-Blank et al., 2014). Samuni-Blank et al. (2014) found similar bacterial communities in the nectar of flowers and the surface of insects that visited the flowers indicating that dispersion of bacteria present in the nectar is not only formed by those present in the air and nectar consumers but also by other vectors such as insects. Anderson et al. (2013) found that many bacteria prevalent in beebread and the crop were also present in floral nectar suggesting frequent horizontal transmission. Also, a symbiotic LAB microbiota within the honey crop of honeybees has been reported (Vásquez et al., 2012). Recently, it was also suggested that floral microbes can mediate plant-bumblebee communication, going their potential beyond microbial effects on nectar chemistry (Russell and Ashman, 2019). On the other hand, Filannino et al. (2018) stated that although reports on endophyte populations of LAB in plants are scarce, advances on plant–microbe interactions have highlighted their importance as a new class of plant growth promoting microbes. To date, *Lb. plantarum* was the only endophytic LAB identified throughout the life cycle of the oregano and wheat plants (Pontonio et al., 2018).

It has been shown that the use of autochthonous LAB strains compared to allochthonous ones as starter cultures is advantageous to enhance the nutritional, sensorial and rheological properties of fermented food products as well as to ensure a prolonged shelf life. LAB selection can be based on pro-technological, sensory, and/or nutritional criteria (Di Cagno et al., 2013, 2015). Additionally, LAB strains belonging to particular niches may present specific metabolic traits as a result of environment adaptation (Siezen and Bachmann, 2008; Endo, 2012). In this regards, plant-associated LAB possess specific enzymes, such as levansucrase, tannase and phytase, and the common feature of producing high amounts of organic acids, such as lactic acid and acetic acid (Tyler et al., 2016). Furthermore, the production of other industrially interesting metabolites, such us aroma compounds, γ-aminobutyric acid, polyols, etc., may also be relevant (Mozzi et al., 2006; Hebert et al., 2008; Abeijón Mukdsi et al., 2009; Dhakal et al., 2012; Quinto et al., 2014; Ruiz Rodríguez et al., 2017a). For instance, mannitol, a compound widely applied in the cosmetic, food, and pharmaceutical industries, is highly produced by certain heterofermentative LAB by reduction of fructose, one of the main sugars present in fruits and vegetables (Endo et al., 2009; Patra et al., 2009, 2011; Carvalheiro et al., 2011; Saha and Racine, 2011; Ortiz et al., 2013; Tyler et al., 2016; Ruiz Rodríguez et al., 2017b).

Several studies on LAB isolation reported a polyphasic approach to achieve a precise microbial identification. However, the choice of appropriate identification methods may depend on certain factors, such as the origin of the sample (clinical, environmental, or food isolates), the number of isolates, and staff qualifications (Moraes et al., 2013). In general, with a few exceptions, a phenotypic test could be proper enough for clinical isolate identification, whereas for food isolates a molecular approach is probably the most sensitive and reliable method (Emerenini et al., 2013; Moraes et al., 2013). Molecular typing has been shown to be useful to group isolates from vegetables and fruits into several clusters for subsequent identification (Papalexandratou et al., 2011a; Di Cagno et al., 2013). One of the most suitable and widely used bacterial identification methods is 16S rRNA gene sequencing. These conserved genes present enough variability to be considered as excellent phylogenetic markers for genus and species level identification (Naeem et al., 2012; Emerenini et al., 2013; Moraes et al., 2013).

The tropical and subtropical areas of the Northern region of Argentina have a large diversity of fruit trees that may be considered as an interesting and rich source of LAB. In this work, we aimed to explore the LAB diversity present on diverse wild fresh fruits and flowers from Northern Argentina as well as to study their technological properties and their ability to produce industrially interesting metabolites.

## Materials and Methods

### Sample Collection

Several units of different fruits (9) and flowers (4) widespread in the Tucumán (27° 00′ 00′′ S, 65° 30′ 00′′ W) province in Northern Argentina were aseptically picked. Ripe wild fruits of guava (pink and yellow varieties), papaya, passion fruit, custard apple, medlar, mulberry, fig, and khaki, and flowers of medlar, passion fruit, custard apple, and papaya (Table 1) were aseptically collected with gloves, put into sterile stomacher bags, and immediately transported to the laboratory for analysis. Sampling was carried out according to the Southern hemisphere seasonal fruit production in the period between April 2013 and April 2014.

**Table 1:** Tropical fruits and flowers from Northern Argentina studied in this work.

**Sample N^**°**^**	**Common name**	**Scientific name**	**Key**	**Collection season**	**Month (year)**
1	Guava	*Psidium guajava*	G	Autumn	April (2013)
2	Papaya	*Carica papaya*	P	Autumn, winter	June, July (2013)
3	Papaya flowers	*Carica papaya*	FP	winter	June, July (2013)
4	Passion fruit	*Passiflora edulis*	My	Winter	July, August (2013)
5	Passion fruit flowers	*Passiflora edulis*	FMy	Autumn	April (2013)
6	Custard apple	*Annona cherimola*	Ch	Autumn	April (2013)
7	Custard apple flowers	*Annona cherimola*	FCh	Autumn	April (2013)
8	Meddlar	*Eriobotrya japonica*	N	Spring	September, October (2013)
9	Meddlar flowers	*Eriobotrya japonica*	FN	Autumn	April (2014)
10	Mulberries	*Morus nigra*	Mr	Spring	October (2013)
11	Fig	*Ficus carica*	H	Summer	January (2014)
12	Khaki	*Diospyros kaki*	Cq	Autumn	April (2014)

### Microbiological Analysis

#### Sample Processing and Isolation of LAB and FLAB From Fruits and Flowers

All samples were processed according to the characteristics of each fruit and flower. For large- and medium-size fruits, such as guava, papaya, passion fruit, custard apple, fig and khaki, separate pools of small portions of the surface and pulp of each unit of fruit were randomly taken. In the case of smaller fruits, such as meddlar and blackberries, as well as flowers, complete units were used. To analyze the LAB and FLAB microbiota present in the samples by direct plating; 90 or 45 mL of peptone water [0.1% (w/v) bacteriological peptone] were added to 10 g of fruit or to 5 g of flower, respectively, and homogenized for 1 min using a stomacher (Stomacher® 400; Seward, Worthing, UK). Appropriate serial dilutions of each suspension were plated onto MRS agar (De Man et al., 1960) for LAB isolation and MRSf agar [MRS agar containing 2% (w/v) of fructose instead of glucose] for FLAB isolation, both supplemented with 0.1 g/L of cycloheximide and 0.1 g/L of sodium azide to inhibit fungi and yeasts, and Gram negative microorganisms, respectively. The total microbial count and coliforms were determined for each sample using plate count agar (PCA; Oxoid Ltd., Basingstoke, Hampshire, UK) and Mac Conkey agar (Britania, Buenos Aires, Argentina), respectively.

Isolation of FLAB was done by culture enrichment according to Endo et al. (2009), with minor modifications. Briefly, 5 mL of FYP broth were added to small pieces of each fruit sample and incubated at 30°C for 24 h. After incubation, 100 μL of the enriched cultures were inoculated into fresh FYP broth and further incubated at 30°C until visible growth detection. Subsequently, serial dilutions of the cultures were plated onto FYP agar containing 5 g/L of CaCO_3_ (this component facilitates LAB detection by formation of a clearance zone around the colonies due to CaCO_3_ hydrolysis by lactic acid).

All plates were incubated at 30°C. MRS and MRSf plates were incubated anaerobically (Anaerobic System AnaeroGen™, Oxoid Ltd.) for 24 to 72 h. FYP and PCA plates were incubated aerobically for 24 to 72 h.

Colonies, randomly selected according to morphological differences (colony size and shape), were picked and purified by streaking on the suitable agar media and further characterized. Representative numbers (30%) of colonies from agar media containing between 30 and 300 CFU/g were picked; when the total colony count was <30, all colonies were analyzed.

Overnight cultures of the isolates were preliminarily assayed for Gram staining, microscopic morphology, and catalase activity. The catalase test was done by suspending bacterial cells in a 3% (v/v) hydrogen peroxide droplet. Gram-positive and catalase-negative cocci and rods were selected as presumptive LAB/FLAB. LAB were stored in a medium containing (g/L): skim milk, 100; yeast extract, 5; glucose, 10; and 10% (v/v) glycerol, while FLAB were stored in nutrient broth containing 20% (v/v) glycerol, both at −20°C.

### Genotypic Characterization

#### Bacterial DNA Extraction

For genomic DNA extraction, two different protocols were used. DNA was either obtained using the commercial DNA extraction kit NucleoSpin®96 Tissue (MACHEREY-NAGEL GmbH & Co. KG, Germany) or extracted according to Pospiech and Neumann (1995) with some amendments. For the latter, three milliliters of stationary phase cultures were centrifuged at 10,000 rpm in an Eppendorf bench top centrifuge for 5 min. Cells were washed with 500 μL of TE buffer [10 mM Tris-HCl (pH 7.5), 10 mM EDTA] and resuspended in 400 mL of STET-lysozyme (15 mg/mL). After holding at 37°C for 2 h, 40 μL (1/10 vol) of 10% sodium dodecyl sulfate (SDS) and 5 μL of proteinase K (15 mg/mL) were added, and the mixture was incubated at 55°C for 2 h. Then, 170 μL (1/3 vol) of 5 M NaCl and 1 vol of chloroform: isoamyl alcohol (24:1) were added to the mixture maintaining it at room temperature for 30 min. After centrifugation at 13,000 rpm for 10 min, the aqueous phase was transferred to another tube, and the DNA was precipitated with isopropanol (1:1 v/v). The precipitate was washed with 500 μL of 70% (v/v) ethanol and centrifuged for 5 min at 13,000 rpm. DNA was dried by evaporating the alcohol and then resuspended in 30 μL of MilliQ water. DNA concentration and purity were spectrophotometrically determined by measuring the optical density (OD) at 260 and 280 nm and determining the OD_260_/OD_280_ ratio (Brown, 1995).

#### Molecular Dereplication of Isolates by rep-PCR Genomic Fingerprinting

To differentiate strains among isolates, microbial dereplication was achieved by rep-PCR fingerprinting (amplification of repetitive bacterial DNA elements through the polymerase chain reaction) of their genomic DNA with the single oligonucleotide primer (GTG)_5_ (5′-GTGGTGGTGGTGGTG-3′) (Versalovic et al., 1994; Gevers et al., 2001), referred to as (GTG)_5_-PCR fingerprinting. The PCR assay mixture (25 μL) consisted of 5 μL buffer [5X Green GoTaq® reaction buffer (Promega, WI, USA)], 1 μL of primer (63 mM), 7.35 μL nuclease-free water, 2.6 μL MgCl_2_ (50 mM), 4 μL bovine serum albumin (BSA; 1 mg/mL), 2.5 μL dimethyl sulfoxide [DMS, 100% (v/v)], 1.25 μL mixture of deoxyribonucleoside triphosphates (dNTPs; dATP, dTTP, dCTP and dGTP, 25 mM), 1 μL template DNA (50 ng/μL) and 0.3 μL *Taq* DNA polymerase (Promega, USA). PCR amplifications were performed with a My Cycler™ thermal cycler (Bio-Rad Laboratories, Inc., Hercules, CA, USA) using the following program: 95°C for 5 min, 30 cycles of 94°C for 1 min, 40°C for 1 min, and 65°C for 8 min, and a final extension step at 65°C for 16 min.

The PCR products were electrophoresed in a 1.5% (w/v) agarose gel (15 × 20 cm) for 16 h at a constant voltage of 55 V in 1x TAE buffer [40 mM Tris-Acetate, 1 mM EDTA (pH 8.0)]. The rep-PCR profiles were visualized after staining with GelRed™ Nucleic Acid Gel Stain (Biotium, Hayward, CA, USA) under UV trans-illuminator (Syngene, Cambridge, UK), and digital image documentation was done using a CCD camera (Canon, Tokyo, Japan). The fingerprints were analyzed by the BioNumerics V4.0 software package (Applied Maths, Sint-Martens- Latem, Belgium). The similarity among digitized profiles was calculated using the Pearson correlation, and an average linkage (UPGMA, unweighted pair group method with arithmetic averages) dendrogram was derived from the profiles obtained.

#### Identification of LAB and FLAB by 16S rRNA Gene Sequencing

Representative LAB and FLAB isolates of all different (GTG)_5_-PCR fingerprint clusters were subjected to sequencing of the variable V1 region of the 16S ribosomal RNA gene using the PLB16 (5 ′AGA GTT TGA TCC TGG CTC AG 3′) and MLB16 (5′TGC GGC GTT TGG GTA CAC AG 3′) primers according to the protocol described by Hebert et al. (2000). The PCR assay mixture (50 μL) consisted of 5 μL 10 × buffer [20 mM Tris-HCl (pH 8.4), 500 mM KCl], 3 μL MgCl_2_ [50 mM], 2 μL of a mixture of dNTPs (dATP, dTTP, dCTP and dGTP, 5 mM), 1 U *Taq* polymerase (Inbio Highway, Buenos Aires, Argentina), 5 μL of each primer [10 μM], 28.7 μL nuclease-free water, and 1 μL of the purified chromosomal DNA (50 ng/μL) as template. PCR amplifications were performed with a My Cycler™ thermal cycler (Bio-Rad Laboratories, Inc.) using the following program: 94°C for 3 min, 30 cycles of 94°C for 30 s, 52°C for 30 s, and 72°C for 45 s, and a final extension step at 72°C for 10 min.

PCR products were electrophoresed in a 1.0% (w/v) agarose gel at 100 V for 45 min in 1x TAE buffer, stained and visualized as described above. The size of DNA fragments (~500 bp) were estimated using a standard 1 kb DNA ladder (1 Kb Plus DNA Ladder, Invitrogen™, Carlsbad, CA, USA). Amplicons were purified by polyethylene glycol precipitation (protocol available at http://gator.biol.sc.edu/ http://labs.mcdb.lsa.umich.edu/labs/olsen/files/PCR.pdf). Nucleotide sequences of purified PCR products were determined at the CERELA-CONICET sequencing facility with an ABI 3130 DNA sequencer (Applied Biosystems, Foster, CA, USA).

#### Sequence Alignments

Identification at species level was achieved by using the BLAST (basic local alignment search tool) program (http://www.ncbi.nlm.nih.gov/BLAST) to compare the obtained rRNA gene sequences with those available at the GenBank database of the National Collection for Biotechnological Information (NCBI; http://www.ncbi.nlm.nih.gov/GenBank/) or Ribosomal Database Project (RDP; http://rdp.cme.msu.edu/) and estimate sequences similarities. For species assignation, a threshold of at least 97% identity with the reference strain in the databases was considered.

### Analysis of Metabolic Properties

#### Mannitol and Organic Acids (Lactic Acid and Acetic Acid) Production

Thirty-eight selected strains belonging to different genera were grown in a formulated fruit simulation medium (FSM) (Ruiz Rodríguez et al., 2017b) with the following composition (g/L): glucose, 10.0; fructose 10.0; sorbitol, 5.0; malic acid, 2.0; MgSO_4_.7H_2_O, 0.2; MnSO_4_.H_2_O, 0.05; K_2_HPO_4_, 1.0; EDTA, 0.1; ammonium citrate, 2.0; vegetable peptone, 10.0; and Tween 80, 1 mL; pH 6.10. Cell-free supernatants (CFS) of 24-h cultures grown in FSM were analyzed for carbohydrate, organic acid, and mannitol content.

The consumption of glucose and fructose and mannitol production by selected strains were determined in the CFS by high-performance anion exchange chromatography (HPAEC) with pulsed amperometric detection, as described by Camu et al. (2007).

Production of lactic acid and acetic acid was determined by high-performance liquid chromatography (HPLC) according to Lefeber et al. (2010), except that an equal volume (500 μL) of acetonitrile was added to the CFS to remove proteins; the mixture was then centrifuged (16,060 × *g* for 15 min) and filtered (0.2 μm Minisart RC4 filters; Sartorius AG) before analysis.

All determinations were performed in triplicate and the mean values and standard deviations of each sample were calculated.

### Aroma Compounds

#### Diacetyl Production

Overnight cultures of the selected strains were inoculated (1%, w/v) in 5 mL of FSM and incubated at 30°C for 48 h. After growth, 2 mL of the culture was supplemented with 1 mL of α-naphthol solution (4%, w/v) and 1 mL of KOH (30%, w/v) and incubated at 30°C for 30 min. Formation of a red/pink ring in the upper part of the cultures indicated diacetyl production (King, 1948). The results were qualitatively defined as negative (-), weak (+), medium (++) or strong (+++) according to the intensity of the color. The strain *Lb. rhamnosus* ATCC 7469 was used as positive control.

#### Ethyl Ester Production

Esterase enzymes, capable of both hydrolyzing and synthesizing esters, play an important role in food flavor development; the balance between synthesis and hydrolysis processes depends on several factors and the specific final product produced in each matrix confers differential organoleptic characteristics to fermented foods (Liu et al., 2004). Fruity ethyl esters can be synthesized by esterification when a fatty acid molecule reacts with ethanol to form an ester and water; this reaction is catalyzed by esterases and its activity is known as reverse esterase activity (REA). First, we evaluated the presence of esterase enzymes in the strains studied by determining the ability to hydrolyze esters. Then, the ability to synthesize esters with particular focus on the fruity ethyl esters was determined in esterase-positive strains.

#### Preparation of Cell-Free Extracts and Esterase Activity

Cells were harvested from 10 mL of FSM cultures after 16 h-incubation at 30°C by centrifugation (8,000 × *g* for 10 min) and washed three times with cold 50 mM potassium phosphate buffer (pH 7.0). The wet cell pellets were mixed with glass beads (150–212 μm, Sigma-Aldrich Chemical Co, MO, USA) in a 1:4:1 (mg cells:μL buffer:mg beads) ratio and subsequently disrupted using a Mini Bed Beater-8 (Biospec Products) for 10 min (with 2 min disruptions in ice every 2 min) at maximum speed. Cell debris and glass beads were removed by centrifugation (12,700 × *g* for 8 min, 4°C), and supernatants were immediately used as cell-free extracts (CFE) for esterase activity determination. Protein content (mg) of CFE were determined by Bradford (Bradford, 1976) with BSA as standard.

Esterase activity (EA_h_) of each CFE was determined on α-naphthyl (α-NA) derivatives (C2, C3, C4, C8, C10, and C12 of carbon atoms) as substrates (Sigma-Aldrich) according to Taboada et al. (2014) with minor changes. Briefly, the reaction mixture contained 18 μL of 100 mM sodium phosphate buffer (pH 7.0), 2 μL of α-NA substrate (10 mM in ethanol), and 20 μL of CFE. After incubation at 30°C for 1 h, the corresponding color was developed by adding 160 μL of Fast Garnet GBC (Sigma-Aldrich) preparation [5 g/L in 100 g/L SDS] and further incubation at room temperature for 15 min. The OD at 560 nm was measured by using a tuneable microplate reader (Versamax TM, Molecular Devices, Sunnyvale, CA, USA). Two standard curves were prepared using α-naphthol. One unit of esterase activity was defined as the amount of α-naphthol released by 1 mL of CFE/min. Specific EA_h_ was defined as units per milligram of protein (U/mg) (Taboada et al., 2014).

#### Ester-Synthesizing Activity

The synthesis of fruity ethyl esters by esterification (also defined as REA) of 2 to 10 C atoms from butanoic acid and hexanoic acid, separately, in the presence of ethanol was studied. Specifically, the esters studied were: acetate-, propionate-, butanoate-, isovalerate-, caproate-, caprylate-, and ethyl caprate. Esters were detected by GC with a flame ionization detector (FID). Ester quantification was done using regression curves of the corresponding standards (R^2^ > 98%). These values were subtracted from the non-enzymatic production and the endogenous ester production, obtained from the substrate and CFE blanks, respectively. Afterwards, the results obtained were corrected by applying the recovery efficiency factor calculated previously, contemplating the product not recovered during the extraction procedure. One enzyme unit (U) was defined as the nmoles of esters formed per mL of enzyme extract per mL of reaction produced in 24 h. The specific activity was expressed as U per mg of total proteins.

Fruit and flower strains representing different LAB genera exhibiting EA_h_ were selected for studying their ester-synthesizing capability. CFE were prepared from 300 mL cultures grown in MRS at 30°C for 16 h, harvested by centrifugation, washed twice with 100 mM phosphate buffer (pH 7.0), and resuspended at 40–60% (w/v) of the same buffer. Cell suspensions were disrupted by three successive passes through a French pressure cell (Thermo Spectronic model FA-078, NJ, USA) at 1,000 psi. Cell debris were removed and the supernatant was used as CFE. The protein content was determined by the Bradford method. Ester-synthesizing activity by esterification was determined by incubation of CFE in an assay mixture containing 100 mM sodium phosphate buffer (pH 7.0), 100 mM ethanol, and 10 mM free fatty acids (butanoic acid or hexanoic acid). After incubation at 30°C for 24 h, esters were extracted with ethyl ether from 1 mL of sample (2:1) and determined by GC [Agilent 6890N (CA, USA), Column HP5 (30 m, 0.32 mm d.i., 0.25 μm), carrier gas (nitrogen gas), detector, (FID)]. Controls lacking substrates or lacking CFE were included. One unit of ester-synthesizing activity was defined as nmoles of ethyl esters formed by 1 mL of CFE in 24 h. Specific ester-synthesizing activity was defined as units per milligram of protein (U/mg) (Abeijón Mukdsi et al., 2009).

### Technological Properties

#### Acidifying Capacity and Growth Rate

Acidification rate and growth kinetics of 80 selected strains were evaluated. Active cultures (16–18 h) of the strains were inoculated [2% (v/v) inoculum] in FSM. After growth, cells were harvested by centrifugation (8,000 × *g* for 10 min at 4°C) and washed twice with 0.1 M sodium phosphate buffer (pH 7.0), resuspended in the same buffer, and the cell suspensions obtained were used as inoculum to evaluate the acidifying capacity (by measuring pH as a function of time), and bacterial growth by turbidimetry (optical density at 600 nm, OD_600_). For both assays, the OD_600_ of cell suspensions was measured and adjusted (Biotraza, model 722, Huida Medical Instruments Co., Jiangsu, China) to obtain microbial cultures with an initial OD_600_ of approximately 0.1.

#### Acidifying Capacity

pH values of culture samples were determined after 0, 2, 4, 6, 8, and 24 h of incubation (pH meter PT-10, Sartorius AG, Gottingen, Germany). Based on the pH curves obtained, the following data were calculated:

**ΔpH8** (pH decrease after 8 h of incubation) = pH (0 h)–pH (8 h);

**ΔpH24** (pH decrease after 24 h of incubation) = pH (0 h) − pH (24 h);

**maximum acidification rate (V**_****max****_, speed of the pH decrease**)**: slope of the curve where the pH decrease is linear and maximum: $\frac{\Delta \text{pH}}{\Delta \text{t}}=\frac{\left(\text{pH}2-\text{pH}1\right)}{\left(\text{t}2-\text{t}1\right)}$.

#### Bacterial Growth

Bacterial growth was evaluated by measuring the OD_600_ (Versamax™ Microplate reader, Molecular Devices, CA, USA) in FSM every 30 min for 16 h. Growth curves were plotted (ln OD_600_ vs. time) and the maximum growth rates (**μ_**max**_**) were calculated as: $\mu_{\max}=\frac{\ln\text{DO}2-\ln\text{DO}1}{\text{t}2-\text{t}1}$.

The results of each parameter were analyzed individually; the Di Rienzo, Guzmán and Casanoves (DGC) test (Di Rienzo et al., 2002) was applied to evaluate statistical significant differences. All results were subjected to principal component analyses (PCA) using the RStudio software (RStudio-Team, 2016).

#### Pectinolytic Activity

Pectinolytic activity was qualitatively examined by pectin depolymerisation in pectin-containing agar media. Pure active cultures were inoculated with 5 μL spots on MRS agar (2%, w/v) without glucose and supplemented with 1% (w/v) citric pectin (Citromax S.A.C.I., Tucumán, Argentina) as the sole added carbon source. Plates were incubated at 30°C for 24 to 72 h. Then, iodine solution was added to detect a clearance zone due to pectin depolymerization after 15 min of staining followed by 15 min of washing with distilled water. The protocol was developed based on the works of Soares et al. (1999); Janani et al. (2011); Varghese et al. (2013); Vidhyasagar et al. (2013).

#### Cinnamoyl Esterase Activity

Cinnamoyl esterase activity was qualitatively evaluated by agar plate assays according to Donaghy et al. (1998) with modifications (Dr. Abeijón Mukdsi, personal communication, CERELA). Ethyl ferulate [EtFA, 2% (v/v) stock solution in methanol] was added to MRS agar media without glucose to a final concentration of 1 g/L. Overnight cultures were inoculated [2% (w/v)] in 5 mL of FSM and incubated at 30°C for 16 h. Grown cultures were centrifuged, washed twice with sodium phosphate buffer (0.1 M, pH 7.0), and resuspended in 5 mL of the same buffer. 50 μL of each suspension was transferred onto 1-cm diameter wells made on EtFA-supplemented agar media. Then, plates were incubated at 30°C for 24–72 h. The strain *Lb. fermentum* ATCC 14932 was used as positive control and the above mentioned buffer was used as negative control. The development of a clear zone around inoculated wells indicated breakdown of EtFA by the corresponding strain.

#### Biogenic Amine Production

Biogenic amine (BA) production was qualitatively evaluated according to Bover-Cid and Holzapfel (1999) with modifications. All strains under study, except for those of the *Enterococcus* species, were used. One strain of *Ec. faecalis* was used as positive control for tyramine production. From an active culture grown in MRS, two successive passages were done in MRSf containing 0.1% (w/v) of L-tyrosine, L-histidine, L-ornithine or L-lysine, to evaluate the production of tyramine, histamine, putrescine or cadaverine, respectively, by induction of the corresponding decarboxylase enzyme. Pyridoxal hydrochloride [0.005% (w/v)] was added as precursor of pyridoxal phosphate, the cofactor of the decarboxylase enzyme. Two successive passages of each active bacterial culture were done in MRSf without the addition of amino acids and were used as negative control. All cultures were incubated at 30°C for 16 h. BA production was determined in solid medium containing 1% (w/v) of each amino acid (tyrosine, histidine, ornithine, or lysine), separately, 0.005% (w/v) pyridoxal hydrochloride, and agar (1.5%, w/v), and with no addition of thiamin. Agar media lacking the presence of amino acids were used as negative control. Each culture was streaked on the five different amino acid-containing agar media and incubated at 30°C for 4 days. The production of BA was monitored by formation of purple color around the producing colonies due to the alkaline nature of BA in the presence of bromocresol purple as indicator.

### Statistical Analysis

All assays were done in triplicate and average values with corresponding standard deviations (SD) are provided. Data were statistically analyzed using the Infostat Statistical Software (Universidad Nacional de Córdoba, Córdoba, Argentina). One-way analysis of variance (ANOVA) with the *post hoc* Tukey's test and DGC test were used to evaluate significant differences among samples. Principal component analysis was performed by using the tools for multivariate data analysis “ade4 package” for the RStudio software.

## Results

### Microbiological Analyses and Isolation of LAB

Selected weather conditions occurring during the fruit and flower sampling months are summarized in Table S1. Data were reported by the Tucumán Aerodrome meteorological station (ICAO: 871210–**SANT**). The majority of the samples were picked during the year 2013, which was particularly dry, registering 61.2% average annual relative humidity and 83 days of rain.

The total microbial counts were between 10^4^ and 10^9^ CFU/g on the fruits assayed and between 10^4^ and 10^6^ CFU/g on the flowers. Coliforms were present in counts of 10^4^-10^8^ CFU/g on the fruits and 10^4^-10^6^ CFU/g on the flowers, whereas LAB were found in lower numbers in the majority of the samples. LAB counts (directly isolated) were in the range of <10^2^-10^4^ CFU/g on the fruits or were not detectable (as in guava, custard apple, meddlar, and mulberries). Conversely, fig and papaya samples presented the highest LAB counts with 10^5^ and 10^7^ CFU/g, respectively. LAB counts in flower samples were in the order of 10^3^ CFU/g with less variable count values than for fruits, the latter ones being strongly dependent on the samples (Table 2).

**Table 2:** Viable colony counts of total bacteria (PCA agar), enterobacteria (Mac Conkey agar), and LAB (MRS and MRSf agar) in fruit and flower samples.

	**log CFU/g**					
**Sample**	**PCA (Total microbial count)**	**Mac Conkey (Enterobacteriaceae)**	**MRS (LAB)**	**MRSf (FLAB)**	**Picked colonies**	**Putative LAB**
Guava	9.0	8.0	3.0	–	135	84
Papaya	7.7	7.7	5.0	–	120	61
Passion fruit	5.7	4.7	<2.0–4.7	4.7	265	85
Passion fruit flowers	5.7	4.7	3.7	–	30	25
Custard apple	4.7	4.0	<2.0	–	78	24
Custard apple flowers	4.0	4.0	3.0	–	8	5
Medlar	7.0	6.0	<2.0	–	89	68
Medlar flowers	>6.0	6.0	3.7	3.7	106	81
Mulberries	4.0	4.0	<2.0	–	72	44
Fig	7.7	4.0	6.7	5.7	317	245
Khaki	6.0	6.0	3.0	3.0	105	103
Total					1325	825

A total of 1,325 colonies were picked from MRS, MRSf, and FYP agar media, derived from all fruit and flower samples; from these, 402 isolates could not be further recovered. Thus, 923 pure isolates were subjected to Gram staining and the catalase test, from which 825 Gram (+) and catalase (–) isolates were selected as putative LAB. The number of presumed LAB was variable among samples; figs being the fruits displaying the highest value (245); in contrast, only 5 isolates were obtained from custard apple flowers (Table 3).

**Table 3:** Distribution of LAB isolates according to their species and the fruit and flower samples.

**Genus**	**Specie**	**Source**											**N^**°**^ different strains/N^**°**^ of isolates**
		**G**	**P**	**My**	**FMy**	**Ch**	**FCh**	**N**	**FN**	**Mr**	**H**	**Cq**	
*Enterococcus*	*casseliflavus*		**16**	**21**	**1**		**1**		**9**				**4/48**
	*casseliflavus/gallinarum*		**1**	**27**	**4**								**3/32**
	*durans*							**3**		**2**	**2**		**1/7**
	*faecalis*				**1**								**1/1**
	*faecium*		**4**					**3**		**2**			**3/9**
	*hirae*		**32**			**2**		**12**				**1**	**4/47**
	*mundtii*					**1**							**1/1**
*Fructobacillus*	*durionis*					**2**					**11**		**2/13**
	*fructosus*										**1**		**1/1**
	*pseudoficulneus*										**1**		**1/1**
	*tropaeoli*					**1**					**129**	**10**	**6/140**
*Lactobacillus*	*brevis*	**13**					**1**						**4/14**
	*plantarum*	**1**											**1/1**
	*rhamnosus*										**43**		**2/43**
*Lactococcus*	*lactis*								**13**		**5**		**3/18**
	*lactis* subsp*. cremoris*		**1**										**1/1**
	*lactis* subsp*. lactis*				**8**				**5**			**4**	**5/17**
*Leuconostoc*	*citreum*											**13**	**2/13**
	*mesenteroides*	**3**						**10**		**26**		**1**	**6/40**
	*mesenteroides* subsp. *dextranicum/mesenteroides*		**1**					**30**					**3/31**
	*mesenteroides* subsp*. mesenteroides*		**1**		**2**					**5**			**6/8**
	*pseudomesenteroides*	**27**		**1**		**3**		**2**	**52**	**9**	**1**	**60**	**24/155**
*Weissella*	*cibaria*				**8**	**10**							**4/18**
	*fabalis*											**2**	**1/2**
	*minor*	**12**											**6/12**
		**56**	**56**	**49**	**24**	**19**	**2**	**60**	**79**	**44**	**193**	**91**	**95/673**

*Each fruit or flower sample were assigned with different colors*.

### Genotypical Analysis. Clustering of Isolates With Rep-PCR, and Identification of Representative Isolates

Putative LAB isolates were subjected to genotyping, using the rep-PCR technique to group those isolates corresponding to clones of the same strain. Once the (GTG)_5_-PCR assays were performed for each isolate, amplified bands were separated by electrophoresis, revealing a wide variety of band profiles among the different samples. Afterwards, (GTG)_5_-PCR fingerprints of the 825 putative LAB isolates were clustered into dendrograms (Figures S1, S2) to have an overview of the diversity of LAB species and strains present on the fruits and flowers assayed. One or more representative isolates of each profile group, representing genotypically different strains of LAB, were subjected to molecular identification. From the total putative LAB, 673 (81.6%) isolates were identified as LAB, whereas 152 (18.4%) isolates belonged to other bacterial groups or were environmental contaminants, such as acetic acid bacteria, staphylococci, etc. From the total LAB isolates, 44.7% (301) were obtained by direct plating of the samples, whereas enrichment cultures allowed the isolation of the remaining 55.3% (372). In this regard, it should be noted that the enrichment steps enabled the isolation of LAB strains from samples for which direct isolation failed, or very few isolates were recovered as in the case of guava, papaya, meddlar, and mulberries (data not shown). Papaya flowers were the only samples from which LAB failed to be isolated.

According to the band profiles obtained by (GTG)_5_-PCR, LAB isolates were distributed into 95 clusters, each representing different LAB strains. Representative isolates from each cluster were identified by comparing their 16S rRNA gene sequences with the available data in the NCBI or RDP databases. The fruits and flowers isolated strains belonged to six LAB genera, namely *Enterococcus, Fructobacillus, Lactobacillus, Lactococcus, Leuconostoc* and *Weissella*, and 21 different species were identified (Table 3). The largest cluster was constituted by 155 isolates belonging to *Leuc. pseudomesenteroides*, which were widely distributed among all samples assayed, except for papaya fruit, passion fruit, and custard apple flowers. The second largest group was formed by 140 fingerprints identified as *F. tropaeoli;* however, this species was only found in 3 different fruits, namely custard apple, fig, and khaki. Analysis of all LAB species showed that the genera *Enterococcus* and *Leuconostoc* were the most widely distributed among the samples studied; nine different *Enterococcus* species were found being this the most diverse genus present on fruits and flowers. On the other hand, *Fructobacillus, Lactobacillus*, and *Weissella* were the least spread genera among all samples. When studying the LAB species diversity present on each type of fruit or flower, it was found that figs and khakis harbored the highest numbers, with 8 and 7 different species, respectively (Table 3).

In those samples where LAB counting was possible, a microbial load analysis of each species was conducted (Table 4). The sample count estimation in MRS and MRSf (when possible) was similar, whereas the species load differed among the samples. For instance, *Ec. casseliflavus* was present in the order of 10^2^ CFU/g on passion fruit flowers and custard apple flowers, 10^3^ CFU/g on papaya and meddlar flowers, and 10^4^ CFU/g on passion fruit. Further, *Leuc. pseudomesenteroides* counts were about 10^2^ CFU/g on passion fruit; 10^2^-10^4^ CFU/g on meddlar flowers, and 10^4^ CFU/g on khaki. Thus, the LAB species count was dependent on the fruit or floral matrix studied.

**Table 4:** Microbial load of each LAB species present in the fruits and flowers assayed, as grown in MRS and MRSf incubated at 30°C for 48 h.

		**CFU/g sample**	
**Sample**	**Species**	**MRS**	**MRSf**
**GUAVA**			
G2	*Lb. brevis*	5.93 10^2^	–
**PASSION FRUIT FLOWER**			
FMy1	*W. cibaria*	2.92 10^3^	–
	*Leuc. mesenteroides* subsp*. mesenteroides*	2.92 10^3^	–
	*Lc. lactis* subsp*. lactis*	7.30 10^3^	–
	*Ec. faecalis*	1.46 10^3^	–
FMy2	*W. cibaria*	1.78 10^4^	–
	*Lc. lactis* subsp*. lactis*	8.88 10^3^	–
FMy3	*Ec. gallinarum/casseliflavus*	1.28 10^3^	–
	*Ec. casseliflavus*	3.20 10^2^	–
**PASSION FRUIT**			
My3	*Ec. casseliflavus*	2.04 10^4^	3.67 10^4^
My4	*Ec. casseliflavus*	1.33 10^4^	–
		2.33 10^3^	–
My11	*Leuc. pseudomesenteroides*	–	1.00 10^2^
	*Ec. casseliflavus*	–	4.00 10^2^
**CUSTARD APPLE FLOWER**			
FCh3	*Lb. Brevis*	4.37 10^2^	–
	*Ec. casseliflavus*	4.37 10^2^	–
**PAPAYA**			
P1	*Ec. casseliflavus*	1.00 10^3^	–
P3	*Leuc. mesenteroides* subsp*. dextranicum/mesenteroides*	1.00 10^2^	–
**MEDLAR FLOWER**			
FN2	*Leuc. pseudomesenteroides*	1.39 10^4^	1.18 10^4^
	*Lc. lactis*	–	6.20 10^3^
FN3	*Leuc. pseudomesenteroides*	2.00 10^2^	5.33 10^2^
	*Lc. lactis*	4.00 10^2^	8.00 10^2^
		1.00 10^3^	–
	*Ec. casseliflavus*	8.00 10^2^	–
		1.00 10^3^	–
**FIG**			
H1	*F. tropaeoli*	5.00 10^2^	1.29 10^3^
	*F. durionis*	2.00 10^2^	2.87 10^2^
		1.00 10^3^	–
H2	*F. tropaeoli*	6.00 10^5^	2.10 10^7^
H3	*F. tropaeoli*	–	3.00 10^5^
	*F. durionis*	6.88 10^5^	–
		1.07 10^6^	–
	*Lc. lactis*	–	3.00 10^5^
		–	3.31 10^5^
	*Lb. rhamnosus*	3.85 10^6^	1.16 10^6^
**KHAKI**			
Cq1	*W. fabalis*	2.00 10^2^	–
	*Leuc. pseudomesenteroides*	1.00 10^4^	5.00 10^3^
		2.10 10^3^	2.20 10^3^
	*Leuc. citreum*	1.00 10^2^	–
	*Ec. hirae*	1.00 10^2^	–
	*F. tropaeoli*	–	4.00 10^2^

### Metabolite Target Analysis

Representative strains of the lactic microbiota present on wild fruits of guava, papaya, passion fruit, custard apple, meddlar, mulberry, fig and khaki, as well as on flowers of medlar, passion fruit, and custard apple from Tucumán were evaluated for their capacity to produce enzymes and compounds of biotechnological interest. Therefore, the metabolites mannitol, lactic acid, acetic acid, and diacetyl, as well as the enzymes cinnamoyl esterase, pectinase, and esterases were determined. These production properties correspond to the criteria normally used for the selection of functional starter cultures to be applied in fruit matrices.

### Organic Acids and Mannitol Production

**Figure 1:**
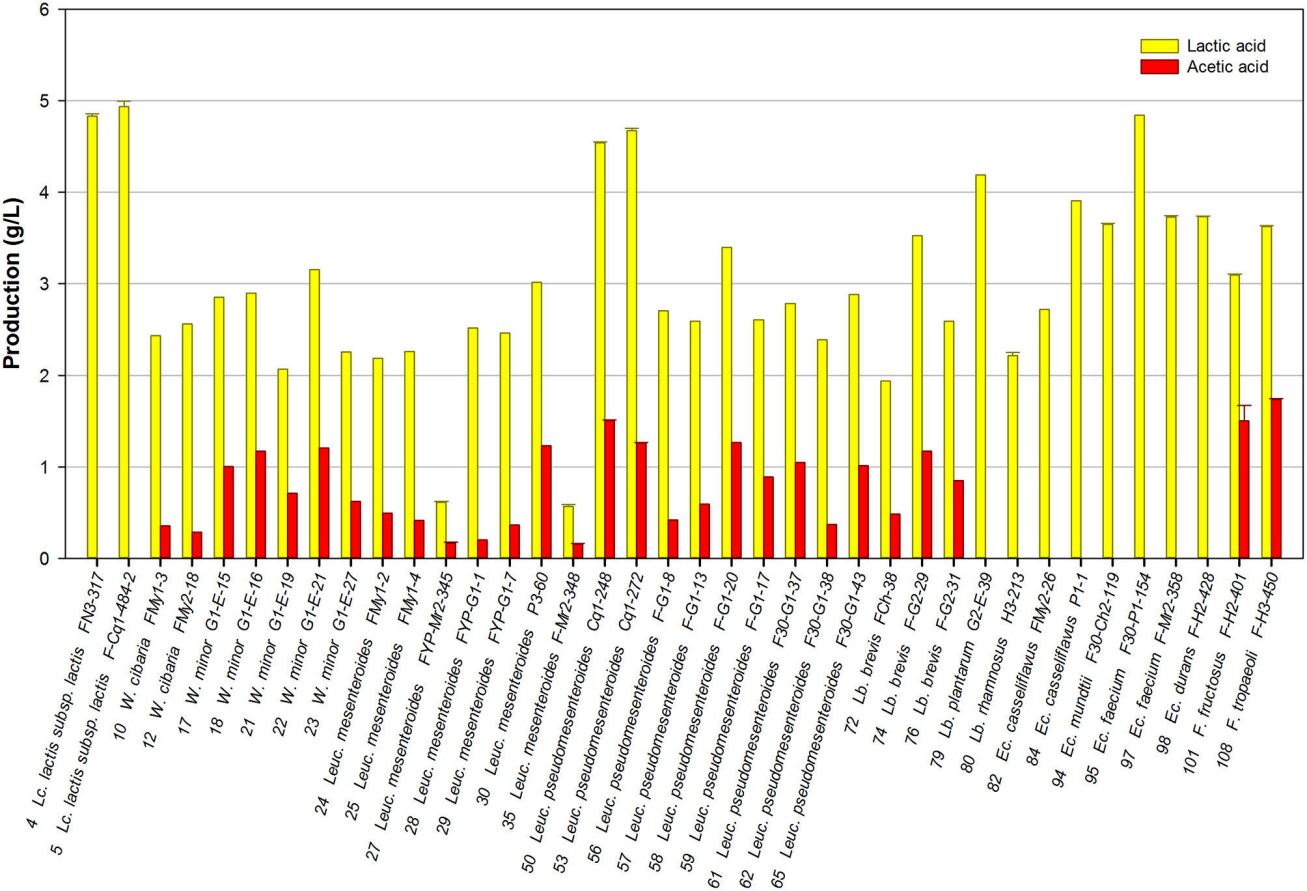
Lactic acid and acetic acid production by selected LAB grown in FSM at 30°C for 24 h.

Thirty-eight representative strains of the isolated LAB species were selected and their ability to produce mannitol was evaluated in the FSM medium. Lactic acid and acetic acid production was simultaneously determined as end-fermentation products. As expected, lactic acid was the main organic acid produced by the strains studied, its concentration being in the range of 0.6–4.9 g/L. The maximal lactic acid production was achieved by strains of the homofermentative species *Lc. lactis*, followed by two *Leuc. pseudomesenteroides*, and one *Enterococcus* strain. Additionally, 28 strains produced acetic acid, though at lower concentrations (0.1–1.7 g/L), among which the *Fructobacillus* and *Leuc. pseudomesenteroides* strains produced the highest amounts (Figure 1). Lactic acid and acetic acid production was variable among the LAB isolates studied.

**Figure 2:**
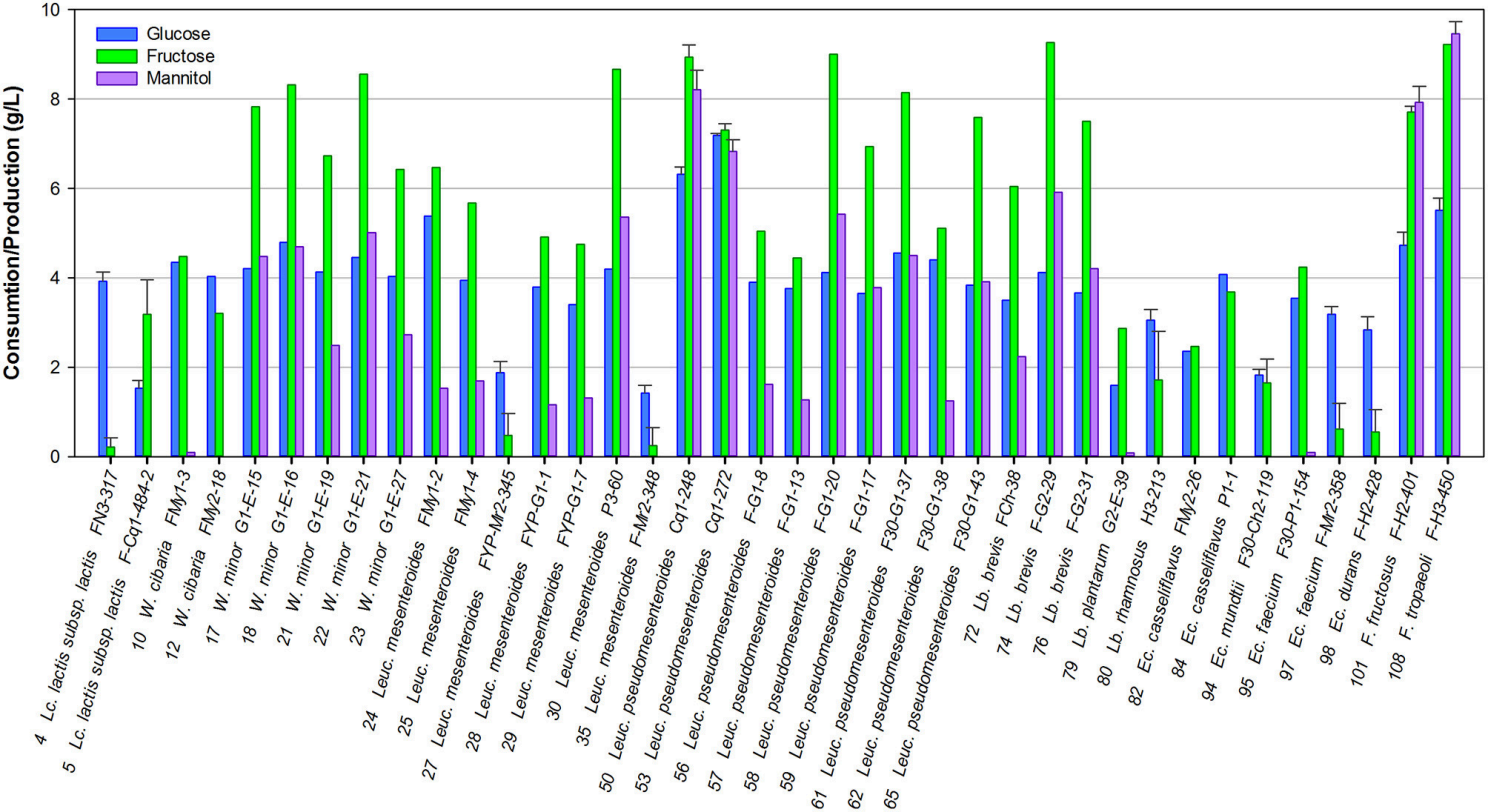
Consumption of sugars and mannitol production by selected LAB grown in FSM at 30°C for 24 h.

Mannitol is a relevant metabolite produced by certain heterofermentative LAB when growing on fructose, one of the most important carbohydrates present in fruits; so, mannitol production was studied in particular. Most of the strains studied were able to consume both monosaccharides present in the culture medium. Only the strains *Lc. lactis* FN3-317, *Leuc. mesenteroides* FYP-My2-345 and FYP-My2-348, *Ec. faecium* FYP-My2-38, and *Ec. durans* FYP-H2-428 grew at the expense of glucose solely, consuming very little fructose (0.22, 0.48, 0, 25, 0.62, and 0.55 g/L, respectively). The rest of the strains consumed equal amounts of both monosaccharides or mainly fructose, which was used for bacterial growth and mannitol production (Figure 2).

Six strains belonging to the heterofermentative genera *Leuconostoc* and *Fructobacillus* produced high concentrations of mannitol (5.2–9.5 g/L). In particular, two strains, namely *F. tropaeoli* FYP-H3-450 (CRL2430, CERELA Culture Collection, Tucumán, Argentina) (Ruiz Rodríguez et al., 2017b) and *F. fructosus* FYP-H2-401, used fructose only as an alternative external electron acceptor, showing 100% mannitol yield in the presence of 10 g/L of fructose. The mannitol-producing phenotype was strain-dependent in most cases (Figure 2).

### Aroma Compounds

#### Diacetyl Production

Fruits and vegetables are fermentable matrices wherein citrate is usually present. Some LAB species can ferment citrate, leading to the biosynthesis of aroma compounds, such as diacetyl, which positively impact the flavor of fermented food products (Smid and Kleerebezem, 2014). Under these considerations, the biosynthesis of 2,3-butanedione in FSM was tested for selected LAB isolates, among which only 23 strains were able to produce diacetyl. According to the intensity of the color, 10 strains of enterococci (5 *Ec. casseliflavus*, 1 *Ec. faecalis*, 1 *Ec. hirae*, 2 *Ec. faecium*, and 1 *Ec. durans*), 1 *Lactobacillus* (*Lb. plantarum* G2-E-39), 3 lactococci (*Lc. lactis* FMy2-21-2, FYP -Cq1-484-2, and FYP P-134-2), 1 *Leuconostoc* (*Leuc. mesenteroides* FYP30-P1-181), and 1 *Fructobacillus* (*F. durionis* H1-167) were weak (+) diacetyl producers, while *Lc. lactis* FN3-308 and FN3-317, *Leuc. pseudomesenteroides* FN3-319 and FN3F-306, and *Ec. faecalis* FMy1-8 showed moderate production (++); *F. fructosus* FYP-H2-401 and *Lb. rhamnosus* H3-213 and H3F-210 displayed strong pink rings (+++), indicating a positive diacetyl reaction (Table 5). Among all diacetyl-producing strains, disregarding the intensity of the fuchsia color generated, the majority corresponded to the genus *Enterococcus* and in second order to *Lactococcus*.

**Table 5:** Technological properties of LAB strains isolated from fruits and flowers from Northern Argentina.

**Microorganism**	**Strain**		**Acidifing and kinetic parameters FSM medium**							**Biogenic amine production**			
			**ΔpH8 ± SD**	**ΔpH24 ± SD**	**V_**max**_ ± SD**	**μ_max_ ± SD**	**Diacetyl production**	**Pectinolytic activity**	**Cinnamoyl esterase activity**	**T**	**H**	**P**	**C**
*Lactococcus lactis* subsp. *lactis*	1	FN3-308	1.92 ± 0.21ª	2.11 ± 0.15ª	−0.55 ± 0.24ª	0.96 ± 0.02^d^	++	++	+	–	–	–	–
	2	FMy2-21-2	1.94 ± 0.18ª	2.17 ± 0.16ª	−0.54 ± 0.18ª	0.97 ± 0.02^d^	+	++	+	–	–	–	–
	4	FN3-317	1.91 ± 0.16ª	2.13 ± 0.15ª	−0.56 ± 0.10ª	0.77 ± 0.01^e^	++	++	+	–	–	–	–
	5	F-Cq1-484-2	1.98 ± 0.16ª	2.22 ± 0.16ª	−0.55 ± 0.03ª	0.90 ± 0.00^d^	+	+	++	–	–	–	–
*Lc. lactis* subsp. *cremoris*	6	F-P-134-2	1.68 ± 0.24ª	1.91 ± 0.21ª	−0.43 ± 0.08^b^	1.25 ± 0.04^b^	+	++	–	+	–	–	–
*Lc. lactis*	9	FN2F-266	1.66 ± 0.24ª	2.03 ± 0.29ª	−0.27 ± 0.16^b^	0.46 ± 0.00^h^	–	+	–	–	–	–	–
*Weissella cibaria*	10	FMy1-3	1.50 ± 0.02ª	1.60 ± 0.19ª	−0.33 ± 0.08^b^	0.95 ± 0.01^d^	–	+	–	–	–	–	–
	11	FMy2-21-1	1.44 ± 0.08ª	1.55 ± 0.27ª	−0.27 ± 0.10^b^	0.97 ± 0.06^d^	–	+	–	–	–	–	–
	12	FMy2-18	1.45 ± 0.02ª	1.56 ± 0.23ª	−0.26 ± 0.09^b^	0.85 ± 0.03^d^	–	++	–	–	–	–	–
*W. fabalis*	14	Cq1-277	0.17 ± 0.00^c^	1.08 ± 0.50^b^	−0.10 ± 0.01^b^	0.67 ± 0.03^e^	–	–	–	–	–	–	–
*W. minor*	16	G1-E-14	0.62 ± 0.16^b^	1.70 ± 0.32ª	−0.12 ± 0.03^b^	0.24 ± 0.02^h^	–	–	–	–	–	–	–
	17	G1-E-15	0.72 ± 0.02^b^	1.88 ± 0.23ª	−0.15 ± 0.03^b^	0.49 ± 0.02^g^	–	–	–	–	–	–	–
	18	G1-E-16	0.62 ± 0.10^b^	1.74 ± 0.08ª	−0.12 ± 0.01^b^	0.55 ± 0.05^f^	–	–	–	–	–	–	–
	21	G1-E-19	1.64 ± 0.38ª	1.92 ± 0.15ª	−0.32 ± 0.07^b^	0.64 ± 0.03^f^	–	–	–	–	–	–	–
	22	G1-E-21	0.80 ± 0.18^b^	1.70 ± 0.22ª	−0.18 ± 0.04^b^	1.29 ± 0.15^b^	–	+	–	–	-	–	–
	23	G1-E-27	0.59 ± 0.04^b^	1.58 ± 0.42ª	−0.15 ± 0.00^b^	0.63 ± 0.07^f^	–	–	–	–	–	–	–
*Leuconostoc mesenteroides* subsp*. mesenteroides*	24	FMy1-2	1.85 ± 0.11ª	1.95 ± 0.19ª	−0.41 ± 0.00^b^	0.64 ± 0.04^f^	–	–	–	–	–	–	–
	25	FMy1-4	1.53 ± 0.00^a^	1.93 ± 0.24ª	−0.28 ± 0.19^b^	0.92 ± 0.06^d^	–	–	+	–	–	–	–
	26	F30-P1-181	1.46 ± 0.13ª	1.61 ± 0.30ª	−0.35 ± 0.01^b^	1.37 ± 0.08^b^	+	+	–	+	–	–	–
	27	F-Mr2-345	1.02 ± 0.15^b^	1.92 ± 0.28ª	−0.21 ± 0.02^b^	1.03 ± 0.12^c^	–	–	–	–	–	–	–
	28	F-G1-1	1.17 ± 1.04^b^	1.95 ± 0.16ª	−0.18 ± 0.03^b^	0.32 ± 0.04^h^	–	–	–	–	–	–	–
	29	F-G1-7	1.49 ± 0.07ª	1.89 ± 0.11ª	−0.35 ± 0.02^b^	0.62 ± 0.05^f^	–	–	–	–	–	–	–
*Leuc. mesenteroides*	30	P3-60	1.81 ± 0.13ª	1.89 ± 0.02ª	−0.40 ± 0.00^b^	0.87 ± 0.05^d^	–	–	–	–	–	–	–
	34	F-Cq1-489	1.92 ± 0.23ª	2.09 ± 0.09ª	−0.39 ± 0.03^b^	0.79 ± 0.00^e^	–	–	–	–	–	–	–
	35	F-Mr2-348	1.01 ± 0.28^b^	2.08 ± 0.22ª	−0.20 ± 0.02^b^	0.75 ± 0.03^e^	–	–	–	–	–	–	–
	37	F-Mr2-343	1.20 ± 0.13^b^	2.05 ± 0.11ª	−0.26 ± 0.03^b^	0.51 ± 0.01^g^	–	–	–	–	–	–	–
	38	F-Mr2-338	1.28 ± 0.19^b^	1.87 ± 0.37ª	−0.22 ± 0.03^b^	0.72 ± 0.03^e^	–	–	–	–	–	–	–
*Leuc. pseudomesenteroides*	39	Cq1-260	1.43 ± 0.78ª	2.00 ± 0.18ª	−0.27 ± 0.21^b^	0.63 ± 0.03^f^	–	–	–	–	–	–	–
	41	F-Mr1-297	1.44 ± 0.36ª	2.02 ± 0.15ª	−0.25 ± 0.07^b^	0.52 ± 0.01^g^	–	+	–	–	–	–	–
	42	F-N1-275	1.61 ± 0.08^b^	1.70 ± 0.13ª	−0.37 ± 0.04^b^	0.70 ± 0.01^e^	–	+	–	+	–	–	–
	43	FN2-284	2.04 ± 0.36^a^	2.18 ± 0.33ª	−0.39 ± 0.12^b^	0.60 ± 0.01^f^	–	+	–	–	–	–	–
	44	Cq1-245	1.72 ± 0.30^a^	1.94 ± 0.23ª	−0.37 ± 0.09^b^	0.46 ± 0.01^g^	–	–	–	–	–	–	–
	45	FN2-296	1.85 ± 0.21ª	2.03 ± 0.13ª	−0.34 ± 0.02^b^	0.56 ± 0.01^f^	–	–	–	–	–	–	–
	46	FN3-319	1.36 ± 0.01^b^	1.66 ± 0.03ª	−0.37 ± 0.00^b^	0.75 ± 0.01^c^	++	–	+	–	–	–	–
	47	FN3F-306	1.35 ± 0.05^b^	1.67 ± 0.03ª	−0.36 ± 0.00^b^	0.72 ± 0.01^c^	++	–	+	–	–	–	–
	48	Cq1-270	1.28 ± 0.41^b^	2.05 ± 0.13ª	−0.37 ± 0.08^b^	0.65 ± 0.00f	–	–	–	–	–	–	–
	49	Cq1F-218	1.55 ± 0.20ª	1.89 ± 0.11ª	−0.34 ± 0.02^b^	0.50 ± 0.01^g^	–	+	–	–	–	–	–
	50	Cq1-248	1.59 ± 0.06ª	1.91 ± 0.08ª	−0.38 ± 0.05^b^	0.65 ± 0.02^f^	–	–	–	–	–	–	–
	51	Cq1-256	1.56 ± 0.06ª	1.89 ± 0.06ª	−0.38 ± 0.02^b^	0.75 ± 0.02^e^	–	+	–	–	–	–	–
	53	Cq1-272	1.51 ± 0.01ª	1.89 ± 0.11ª	−0.35 ± 0.01^b^	0.61 ± 0.10^f^	–	+	–	–	–	–	–
	56	F-G1-8	1.61 ± 0.02ª	1.91 ± 0.01ª	−0.36 ± 0.01^b^	0.58 ± 0.01^f^	–	+	–	–	–	–	–
	57	F-G1-13	1.35 ± 0.34^b^	1.77 ± 0.03ª	−0.24 ± 0.15^b^	1.58 ± 0.01ª	–	+	–	–	–	–	–
	58	F-G1-20	1.03 ± 0.00^b^	1.77 ± 0.00^a^	−0.31 ± 0.04^b^	0.71 ± 0.01^e^	–	+	–	–	–	–	–
	59	F-G1-17	1.25 ± 0.01^b^	1.75 ± 0.14ª	−0.29 ± 0.00^b^	0.58 ± 0.01^f^	–	+	–	–	–	–	–
	60	F-G1-19	0.63 ± 0.00^b^	1.86 ± 0.00^a^	−0.17 ± 0.02^b^	0.86 ± 0.05^d^	–	+	–	–	–	–	–
	61	F30-G1-37	1.57 ± 0.05ª	1.85 ± 0.17ª	−0.31 ± 0.04^b^	1.04 ± 0.02^c^	–	++	–	–	–	–	–
	62	F30-G1-38	1.10 ± 0.54ª	1.73 ± 0.09ª	−0.43 ± 0.10^b^	0.71 ± 0.03^e^	–	+	–	–	–	–	–
	65	F30-G1-43	0.62 ± 0.25^b^	1.70 ± 0.16ª	−0.18 ± 0.27^b^	0.52 ± 0.01^g^	–	+	–	–	–	–	–
	69	F-G2-25	0.69 ± 0.13^b^	1.86 ± 0.07ª	−0.28 ± 0.01^b^	0.52 ± 0.01^g^	–	–	–	–	–	–	–
*Leuc. citreum*	70	F-Cq1-501	1.85 ± 0.04ª	1.95 ± 0.03ª	−0.33 ± 0.01^b^	0.79 ± 0.00^e^	–	–	–	–	–	–	–
	71	F-Cq1-496	1.77 ± 0.12ª	1.86 ± 0.08ª	−0.37 ± 0.03^b^	0.79 ± 0.00^e^	–	–	–	–	–	–	–
*Lactobacillus brevis*	72	FCh3-38	0.83 ± 0.34^b^	1.77 ± 0.07ª	−0.16 ± 0.02^b^	0.34 ± 0.01^h^	–	+	–	–	–	–	–
	73	G2-E-50	1.58 ± 0.45ª	2.35 ± 0.06ª	−0.31 ± 0.06^b^	0.40 ± 0.00^g^	-	–	–	–	–	–	–
	74	F-G2-29	0.90 ± 0.04^b^	1.82 ± 0.23ª	−0.19 ± 0.01^b^	0.42 ± 0.01^g^	–	+	–	–	–	–	–
	76	F-G2-31	0.97 ± 0.35^b^	1.94 ± 0.11ª	−0.18 ± 0.02^b^	0.46 ± 0.01^g^	–	+	–	–	–	–	–
*Lb. plantarum*	79	G2-E-39	0.73 ± 0.45^b^	2.18 ± 0.18ª	−0.21 ± 0.11b	0.54 ± 0.02^g^	+	–	+	–	–	–	–
*Lb. rhamnosus*	80	H3-213	0.67 ± 0.19^b^	1.96 ± 0.27ª	−0.16 ± 0.03^b^	0.44 ± 0.08^g^	+++	+	–	–	–	–	–
	81	H3F-210	0.59 ± 0.18^b^	2.14 ± 0.15ª	−0.15 ± 0.04^b^	0.41 ± 0.04^g^	+++	+	–	–	–	–	–
*Fructobacillus durionis*	100	H1-167	1.50 ± 0.01ª	1.62 ± 0.13ª	−0.28 ± 0.07^b^	0.77 ± 0.02^e^	+	–	–	–	–	–	–
*F. fructosus*	101	F-H2-401	1.55 ± 0.18ª	1.60 ± 0.10ª	−0.37 ± 0.05^b^	0.68 ± 0.07^e^	+++	–	–	–	–	–	–
*F. pseudoficulneus*	102	F-H3-468	1.59 ± 0.20ª	1.64 ± 0.12ª	−0.45 ± 0.12^b^	0.86 ± 0.04^d^	–	–	–	–	–	–	–
*F. tropaeoli*	103	F30-Ch2-116	1.53 ± 0.01ª	1.69 ± 0.00^a^	−0.46 ± 0.13^b^	0.73 ± 0.00^e^	–	–	–	–	–	–	–
	104	F-H1-384	1.56 ± 0.26ª	1.67 ± 0.08ª	−0.40 ± 0.09^b^	0.90 ± 0.00^d^	–	–	–	–	–	–	–
	105	H2-200	1.76 ± 0.07ª	1.82 ± 0.11ª	−0.42 ± 0.03^b^	0.66 ± 0.01^f^	–	–	–	–	–	–	–
	106	H1F-130	1.77 ± 0.11ª	1.78 ± 0.08ª	−0.43 ± 0.09^b^	0.64 ± 0.00^f^	–	–	–	–	–	–	–
	107	Cq1F-246	1.74 ± 0.32ª	1.80 ± 0.20ª	−0.40 ± 0.10^b^	0.82 ± 0.02^d^	–	+	–	–	–	–	–
	108	F-H3–450	1.69 ± 0.04ª	1.73 ± 0.08ª	−0.39 ± 0.18^b^	0.53 ± 0.02^g^	–	–	–	–	–	–	–
*Ec. casseliflavus /gallinarum*	82	FMy3-26	n.d.	n.d.	n.d.	n.d.	–	+	+	n.d.	n.d.	n.d.	n.d.
	83	F-My4-243	n.d.	n.d.	n.d.	n.d.	+	+	+	n.d.	n.d.	n.d.	n.d.
	84	P1-1	n.d.	n.d.	n.d.	n.d.	+	–	+	n.d.	n.d.	n.d.	n.d.
*Ec. casseliflavus*	85	FN3-310	n.d.	n.d.	n.d.	n.d.	+	+	–	n.d.	n.d.	n.d.	n.d.
	86	F-P1-150	n.d.	n.d.	n.d.	n.d.	+	+	–	n.d.	n.d.	n.d.	n.d.
	87	F30-P1-182	n.d.	n.d.	n.d.	n.d.	+	+	–	n.d.	n.d.	n.d.	n.d.
	88	FCh3-31	n.d.	n.d.	n.d.	n.d.	–	-	+	n.d.	n.d.	n.d.	n.d.
*Ec. faecalis*	89	FMy1-8	n.d.	n.d.	n.d.	n.d.	++	+	–	+	–	–	–
*Ec. hirae*	90	F-Ch2-102	n.d.	n.d.	n.d.	n.d.	+	+	–	n.d.	n.d.	n.d.	n.d.
	91	F-N1-266	n.d.	n.d.	n.d.	n.d.	–	+	–	n.d.	n.d.	n.d.	n.d.
	92	F30-P1-160	n.d.	n.d.	n.d.	n.d.	–	+	–	n.d.	n.d.	n.d.	n.d.
	93	F-N1-272	n.d.	n.d.	n.d.	n.d.	–	+	–	n.d.	n.d.	n.d.	n.d.
*Ec. mundtii*	94	F30-Ch2-119	n.d.	n.d.	n.d.	n.d.	–	–	–	n.d.	n.d.	n.d.	n.d.
*Ec. faecium*	95	F30-P1-154	n.d.	n.d.	n.d.	n.d.	+	+	–	n.d.	n.d.	n.d.	n.d.
	96	F-N1-291	n.d.	n.d.	n.d.	n.d.	+	+	–	n.d.	n.d.	n.d.	n.d.
	97	F-Mr2-358	n.d.	n.d.	n.d.	n.d.	–	–	–	n.d.	n.d.	n.d.	n.d.
*Ec. durans*	98	F-H2-428	n.d.	n.d.	n.d.	n.d.	+	–	–	n.d.	n.d.	n.d.	n.d.

***T:** tyramine, **H:** histidine, **P:** putrescine, **C:** cadaverine*.

*Mean values (± standard deviation) within the same column followed by different superscript letters are significantly different (p < 0.05) by DGC test*.

#### Production of Fruity Esters

The specific esterase activity of hydrolysis (EA_h_) of α-naphthyl derivatives (α-NA) with carbon chains of C2, C3, C4, C8, C10, and C12 as carbon substrates using CFE was studied. Then, the REA for fruity ethyl ester biosynthesis was determined for selected strains.

**Figure 3:**
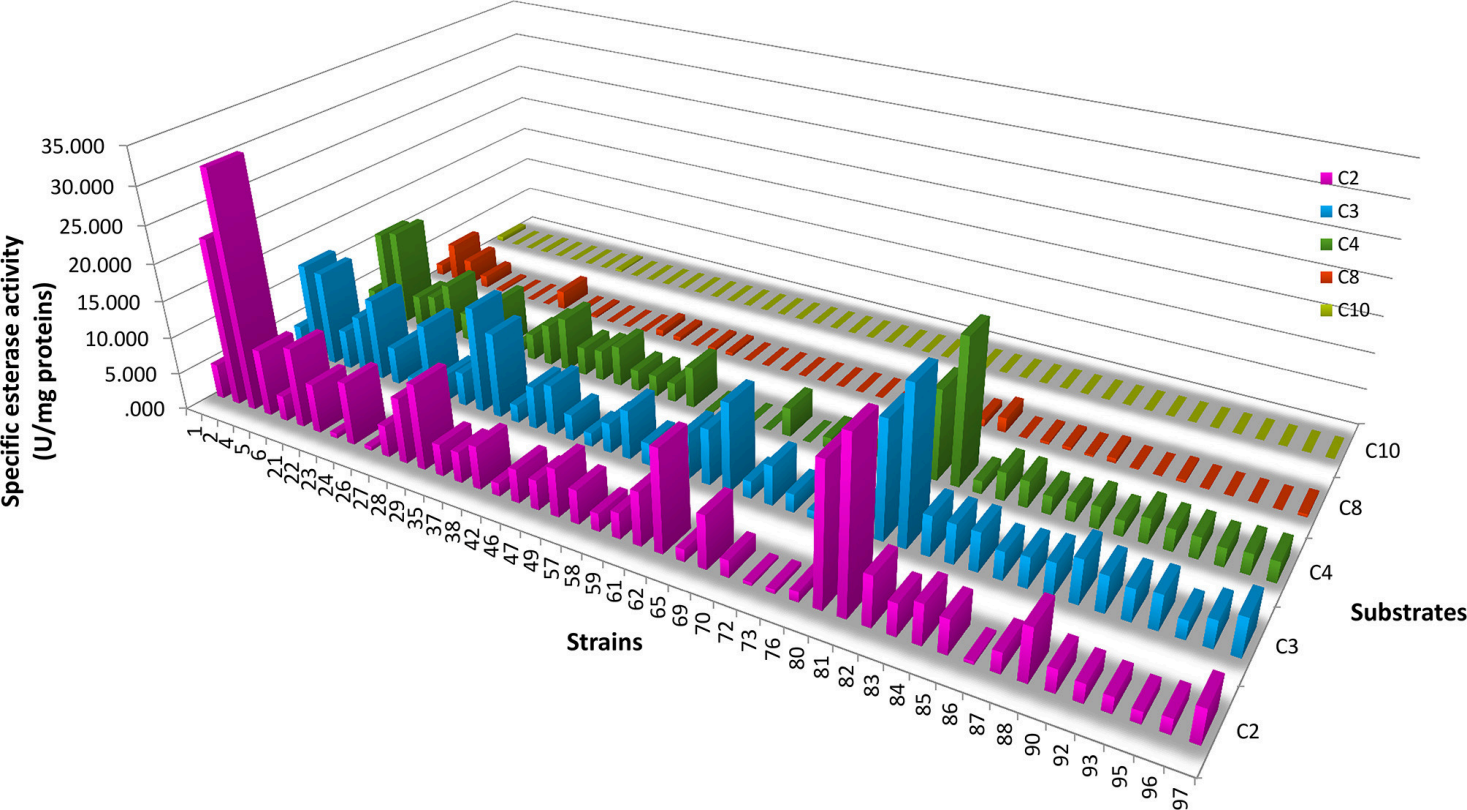
Esterase activity of hydrolysis of α-naphthyl derivatives (carbon chains of C2, C3, C4, C8, and C10) by selected LAB strains. Specific esterase activity values are indicated for each substrate. The strain numbers correspond to the codes given in Table 5.

From the total LAB isolates, 45 LAB strains belonging to 5 genera (*Lactococcus, Weissella, Leuconostoc, Lactobacillus*, and *Enterococcus*) showed EA_h_ with at least two α-NA derivatives (Figure 3); all strains were active against α-NA-C2 and α-NA-C3 and none against α-NA-C12. The EA_h_ on α-NA-C2, α-NA-C3, α-NA-C4 and α-NA-C8 was widely distributed among the LAB strains studied, whereas only *Lc. lactis* subsp*. lactis* F-Cq1-484-2, *Leuc. mesenteroides* FMy1-2 and *Lc. lactis* subsp. *lactis* FN3-308 hydrolyzed α-NA-C10. *Lc. lactis* FN3-317 showed the highest EA_h_ value (32.38 ± 8.45 U/mg) on α-NA-C2, whereas *Lb. rhamnosus* H3F-210 showed the highest values on α-NA-C3 and α-NA-C4 (20.89 ± 6.49 and 19.76 ± 2.26 U/mg, respectively). On the other hand, all strains of *Lb. brevis* showed low EA_h_ values, while the behavior of all enterococci was similar with respect to hydrolysis of α-NA-C3 and α-NA-C4 substrates.

Based on the results obtained, 8 strains were selected to further study the production of ethyl fruity esters. In general, varied activity values (0.97–22.43 U/mg) of ethyl ester biosynthesis were detected, being both strain- and substrate-dependent (Table 6). All strains produced ethyl acetate in diverse concentrations (1.55–28.75 nmol/mg protein) when using butanoic acid as substrate. Moreover, most strains produced at least one additional ester (ethyl propionate and/or ethyl butanoate) from the above mentioned substrate, with the exception for *W. minor* G1-E-19. Also, 4 strains could produce esters from hexanoic acid (the 2 strains of *Lc. lactis, Leuc. pseudomesenteroides* F30-G1-38, and *Lb. rhamnosus* H3F-210). High levels of short-chain ethyl esters were found, namely EtC2, EtC3, and EtC4 from butanoic acid and EtC2 and EtC3 from hexanoic acid, whereas no formation of esters of higher carbon atoms (EtC5, EtC6, EtC8, and EtC10) were detected with any of the substrates used. The highest REA was found for the strains *Lb. rhamnosus* H3F-210 and *W. minor* G1-E-19, with values of 22.43 ± 1.70 U/mg protein for ethyl butanoate and 17.97 ± 1.20 U/mg protein for ethyl acetate, respectively. The latter compound was the only ester formed by the *W. minor* strain that was also the only one producing a single type of ester. The highest ethyl propionate activity corresponded to *Lc. lactis* FMy2-21-2 (4.95 ± 0.83 U/mg protein), this value being 4 to 5 times lower than the maximum values detected for the biosynthesis of ethyl acetate and ethyl butanoate. *Lc. lactis* FN3-317 was the only strain that produced the 3 types of ethyl esters detected while *Lb. brevis* F-G2-31 presented remarkable values of ethyl acetate and ethyl butanoate activity (11.33 ± 1.05 and 4.86 ± 0.8 U/mg protein, respectively), despite being the strain with the lowest EA_h_ among the selected strains (1.35 ± 0.72 and 1.23 ± 0.06 U/mg protein on α-NA-C2 and α-NA-C4, respectively).

**Table 6:** Specific activity for the production of fruity ethyl esters by selected LAB strains.

**Strain**	**Specific ethyl ester-producing activity (U/mg protein)**					
	**Butanoic acid + Ethanol**			**Hexanoic acid + Ethanol**		
	**EtC2**	**EtC3**	**EtC4**	**EtC2**	**EtC3**	**EtC4**
2 *Lc. lactis* FMy2-21-2	1.89 ± 0.76^e^	4.95 ± 0.83^a^	n.d.	n.d.	2.92 ± 0.64^a^	n.d.
4 *Lc. lactis* FN3-317	3.42 ± 0.72	4.07 ± 0.75^a^	7.85 ± 0.95^b^	n.d.	3.71 ± 0.70^a^	n.d.
21 *W. minor* G1-E-19	17.97 ± 1.20^a^	n.d.	n.d.	n.d.	n.d.	n.d.
24 *Leuc. mesenteroides* FMy-1-2	4.38 ± 0.83^d^	n.d.	1.94 ± 0.60^d^	n.d.	n.d.	n.d.
29 *Leuc. mesenteroides* F-G1-7	0.97 ± 0.35^e^	2.61 ± 0.60^b^	n.d.	n.d.	n.d.	n.d.
62 *Leuc. pseudomesenteroides* F30-G1-38	6.41 ± 0.90^c^	n.d.	2.35 ± 0.75^d^	8.29 ± 01.10^a^	n.d.	n.d.
76 *Lb. brevis* F-G2-31	11.33 ± 1.05^b^	n.d.	4.86 ± 0.80^c^	n.d.	n.d.	n.d.
81 *Lb. rhamnosus* H3F-210	2.88 ± 0.85^e^	n.d.	22.43 ± 1.70^a^	8.77 ± 1.20^a^	n.d.	n.d.

*Only the results for the esters of fatty acids with 2 to 4 C atoms are shown (no esters of fatty acids with 5 to 10 C were detected). **EtC2**, ethyl acetate; **EtC3**, ethyl propionate; **EtC4**, ethyl butanoate; **n.d**., not detected. Mean values (± standard deviation) within the same column followed by different superscript letters are significantly different (p < 0.05) by Tukey test*.

**Figure 4:**
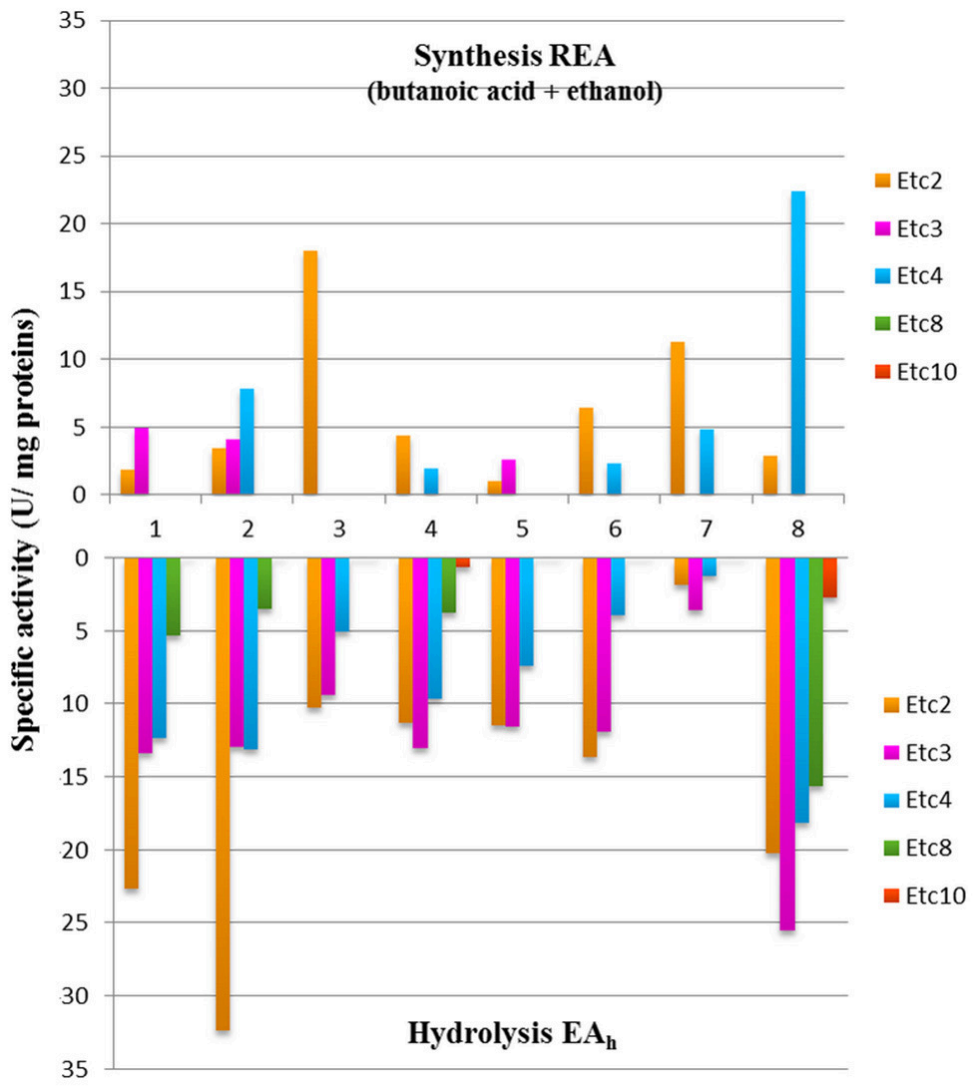
Esterase activity of biosynthesis and hydrolysis of ethyl esters by 8 LAB strains. 1*, Lc. lactis* FMy2-21-2; 2, *Lc. lactis* FN3-317; 3, *W. minor* G1-E-19; 4, *Leuc. mesenteroides* FMy-1-2; 5, *Leuc. mesenteroides* F-G1-7; 6, *Leuc. pseudomesenteroides* F30-G1-38; 7, *Lb. brevis* F-G2-31; and 8, *Lb. rhamnosus* H3F-210.

Remarkably, when comparing the EA_h_ and REA values of the strains capable of producing ethyl esters, the ability to hydrolyze esters of a certain chain length did not always correspond with the ability to produce ethyl esters of the same length (Figure 4).

### Properties of Technological Interest

#### Acidifying Capacity and Growth Rate

LAB are characterized by rapid acidification during growth due to the production of organic acids, such as lactic acid, acetic acid, and formic acid, depending on their ability to ferment carbohydrates. The acidifying capacity of 67 LAB strains from tropical fruits and flowers was evaluated by determining their pH curves during a 24 h-period, considering that the established time of fruit and vegetable fermentation is between 15 and 24 h (Di Cagno et al., 2013). From the pH values obtained, ΔpH8, ΔpH24, and maximum acidification rates (V_max_) were calculated_**.**_ Likewise, microbial growth (OD_600_ and maximum specific growth rate, μ_max_) were determined (Table 5).

The maximum ΔpH8 and ΔpH24 values in FSM corresponded to *Leuc. pseudomesenteroides* FN2-284 and *Lb. brevis* G2-E-50, respectively while the maximum value of V_max_ corresponded to *Lc. lactis* subsp. *lactis* FN3-317. The highest μ_max_ value corresponded to *Leuc. pseudomesenteroides* F-G1-13. It is interesting to highlight that in some cases strains belonging to the same species showed a similar behavior; for example, all strains of *Lc. lactis* subsp. *lactis* showed high acidification and growth rate values. Most strains of *W. minor* and *Lactobacillus* showed low acidifying capacity and low μ_max_ values. For the remaining species, the acidifying behavior was strain-dependent. It is important to emphasize that out of 67 strains assayed, 66 (except for *W. fabalis* Cq1-277) reached a pH value ≤4.5 within 24 h of fermentation, which is an essential requirement for fruit fermentation. Additionally, 10 strains decreased the pH to 4.5 in only 6 h of incubation, whereas 26 did so after 8 h (data not shown).

As expected, a positive correlation between the acidifying capacity and the growth rate of the strains was found.

As maximum V_max_ and μ_max_ values were strain-dependent, the existence of statistically significant differences between the values of each parameter of all strains was studied. In general, limited statistically significant variability between the ΔpH and V_max_ values was found, unlike the μ_max_ values, which were more diverse, allowing grouping of the strains into 8 categories significantly different among each other (a-h, Table 5).

**Figure 5:**
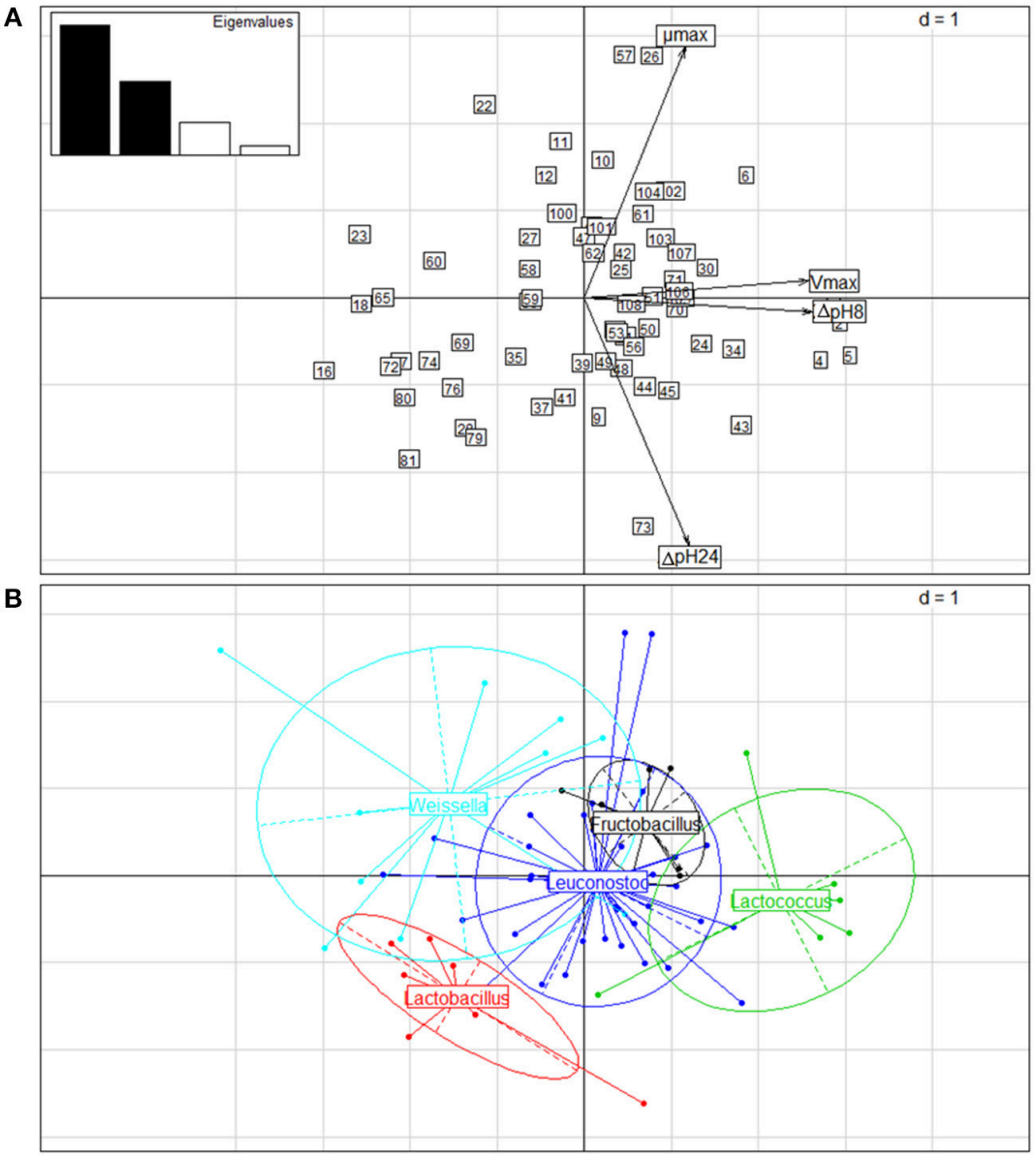
Principal component analysis (PCA) of the kinetic parameters of 67 LAB strains isolated from wild fruits and flowers in Northern Argentina. **(A)** Biplot of PCA obtained considering ΔpH8, ΔpH24, V_max_ and μ_max_ of the strains grown in FSM medium at 30°C for 24 h. Arrows correspond to eigenvectors for the kinetic parameters. The numbers of the strains correspond to the codes presented in Table 5. **(B)** Biplot of the strain distribution obtained, considering the genus as classification factor.

The results of the acidifying capacity and growth rates of the strains were also subjected to PCA. Two principal components (PC) accounted for 82.79% of the total variance. Figure 5 shows the biplot of the PCA for the first (PC1) and the second (PC2) PC, which explained 52.86 and 29.93% of the total variance, respectively. PC1 (vertical axis) separated strains displaying the highest values of ΔpH8 and V_max_ to the right, whereas to the left samples appeared with low variable values. PC2 (horizontal axis) separated upwards those strains with higher μ_max_ and downwards those with higher ΔpH24 values (Figure 5A). Considering the genus as classification factor (Figure 5B), the *Lactococcus* strains presented ΔpH8 and V_max_ values higher than the rest of the genera, while the genus *Lactobacillus* differed from the rest throughout horizontal axis, showing higher ΔpH24 values but lower μ_max_ values than the rest. The genera *Leuconostoc, Weissella*, and *Fructobacillus* could not be clearly differentiated from each other using these 2 PC analyses, probably due to the close phylogenetic relationship among these three genera belonging to the Leuconostocaceae family.

#### Pectinolytic Activity

Pectinases are enzymes that break down pectin substances that are used in the industry to increase the yield and clarity of fruit juices. Pectic polysaccharides are normal components of plant tissues, thus LAB present on fruits and vegetable surfaces could harbor pectinolytic activity as a niche adaptation feature. From the total evaluated strains (43), 51.2% showed a clearance zone of pectin depolymerization around the assayed spots. The largest hydrolysis halos of citrus pectin were found around growing colonies of all lactococci, particularly for the strain *Lc. lactis* subsp. *lactis* FN3-317. Among the enterococci, 12 strains hydrolyzed citrus pectin, whereas almost all lactobacilli, except for *Lb. brevis* G2-E-50 and *Lb. plantarum* G2-E-39, and 14 strains of *Leuc. pseudomesenteroides* exhibited pectinase activity. On the other hand, this property was generally absent in the *Fructobacillus* species (except for *F. tropaeoli* Cq1F-246), all strains of *Leuc. mesenteroides* (with the exception of F30-P1-181) and almost all *Weissella* isolates, among which *W. minor* G1-E-21 and 3 strains of *W. cibaria* (FMy1-3, FMy2-21-1 and FMy2- 18) were pectinolytic strains (Table 5).

#### Cinnamoyl Esterase Activity

Some LAB show esterase activity on plant phenolic compounds (esters of hydroxycinnamic acids), releasing the corresponding acid whose beneficial properties for human health have been widely studied. The capacity of the strains assayed to hydrolyze ethyl ferulate was qualitatively evaluated. From the total LAB strains studied, only 12 strains showed hydrolysis of ethyl ferulate, although weak activity was found as compared to the control strain *Lb. fermentum* ATCC 14932. Four *Lc. lactis* strains, 1 *W. minor*, 1 *Leuc. mesenteroides*, 2 *Leuc. pseudomesenteroides*, 1 *Lb. plantarum*, and 4 *Ec. casseliflavus* showed small halos of positive cinnamoyl esterase activity, among which *Lc. lactis* F-Cq1-484-2 presented a larger and sharper halo (Table 5).

#### Production of Biogenic Amines

The intake of foods with high levels of BA may cause toxicological problems. In some fermented foods, either spontaneously or through the use of starter cultures, BA may be present as result of microbial metabolism. Thus, the inclusion of BA-producing strains in starter cultures should be avoided. The ability of 67 LAB strains to produce BA, particularly histamine, tyramine, putrescine, and cadaverine, was studied (Table 5). All *Enterococcus* strains were excluded because they frequently produce BA and carry virulence factors, and their intentional use is highly questioned in food processing. However, a tyramine-producing *Ec. faecalis* strain was used as positive control (Bover-Cid and Holzapfel, 1999).

Among the 67 strains evaluated, only 3 produced tyramine, which was visualized by purple coloration around colonies on tyrosine-containing agar media. The capacity to produce BA was rarely widespread among the flower- and fruit-origin strains studied.

As the number of strains belonging to each studied genus was substantially different, the percentage of strains of the same genus capable of displaying a specific property should be considered. Thus, it is important to highlight that would be necessary to increase the number of strains of a particular genus (i.e., *Lactococcus* in this study) to be able to conclude about how frequent a particular characteristic is detected.

## Discussion

Microbiological studies of 12 types of wild fruits and flowers disseminated in the province of Tucumán, Argentina, revealed that the estimated total microbial population fluctuated between 10^5^ and 10^9^ CFU/g and 10^4^ and 10^5^ CFU/g on fruits and flowers, respectively, in coincidence with other studies indicating values between 10^5^ and 10^7^ CFU/g on fruits and vegetables (Di Cagno et al., 2010, 2015). Regarding LAB populations present in this type of niches, the present study showed that colony counts obtained by direct isolation were variable and dependent on the samples, being in general between <10^2^-10^4^ CFU/g, or not detectable, such as in fruits as passion fruit, custard apple, medlar, and mulberries. On the contrary, high LAB counts were recorded in the order of 10^7^ and 10^5^ CFU/g in fig and papaya samples, respectively, these values being similar to those reported by other authors for different fruits, whereas Fessard et al. (2016) reported the same counts for papaya. Di Cagno et al. (2010, 2011a) found that mesophilic LAB populations on blackberries, prunes, kiwis, and papayas ranged between 10^2^ and 10^4^ CFU/g, whereas on pineapple the counts were higher (10^4^-10^5^ CFU/g). Bae et al. (2006) found that LAB were present in 10^2^ and 10^4^ CFU/mL in grape juice homogenates, whereas counts in *masau* tropical fruit were 10^3^ CFU/g (Nyanga et al., 2007).

From the 12 wild fruits and flowers studied, 673 isolates were confirmed as LAB; the isolated LAB/sample average ratio (673/12) being higher than for most other works available in the literature for this types of matrices, although the isolation protocols were similar; some differences occurred in the sample type and treatments applied (commercial or wild, washed or unwashed, direct isolation or by culture enrichment) in each case (Bae et al., 2006; Trias Mansilla et al., 2008; Chen et al., 2010; Naeem et al., 2012). Bae et al. (2006) only found LAB by direct plating in 4 out of 43 batches of grape homogenates studied and could isolate 160 LAB mainly by culture enrichment. Chen et al. (2010) isolated 88 LAB from blackberry samples from 5 different farms in Taiwan, whereas Di Cagno et al. (2010) isolated 104 LAB from pineapple. While Emerenini et al. (2013) obtained 105 LAB isolates from different fruits (citrus, bananas, tomatoes) and vegetables (pumpkin and green vegetables), Garcia et al. (2016) isolated 50 LAB from samples of Barbados cherry, mango, soursop, and strawberry.

Strain biodiversity in the fruit and flower samples assayed was estimated according to the band profiles obtained by using the (GTG)_5_-PCR genotyping technique. The rep-PCR technique has been recognized as a simple technique, with high discriminatory power, low cost, suitable for handling large numbers of strains, and for classifying a wide range of Gram (+) and Gram (–) bacteria at species, subspecies, and/or strain level (Gevers et al., 2001). Rep-PCR has been used for typing LAB from different origins by several authors (Gevers et al., 2001; Ouadghiri et al., 2005; Vasilopoulos et al., 2008; Sengun et al., 2009; Papalexandratou et al., 2011b; Perin and Nero, 2014; Šalomskiene et al., 2015; Sáez et al., 2017). In this work, 673 isolates were grouped into 95 different clusters according to their band profiles, indicating higher species and strain diversity of LAB in the samples examined than that found in other fruits and vegetables (Bae et al., 2006; Chen et al., 2010; Di Cagno et al., 2010; Askari et al., 2012; Emerenini et al., 2013; Wu et al., 2014); (Verón et al., 2017).

The isolated LAB strains belonged to 21 different species of the genera *Enterococcus, Fructobacillus, Lactobacillus, Lactococcus, Leuconostoc*, and *Weissella*. The genera *Fructobacillus, Lactobacillus*, and *Weissella* were less spread among the samples studied, *Enterococcus* and *Leuconostoc* being the most widely distributed genera. Many of the LAB species isolated in this work are typically associated with the plant environment. *Ec. faecalis, Ec. faecium, Ec.mundtii*, and *Ec. casseliflavus* are commonly plant-associated species; while *Ec. durans* and *Ec. hirae* have been found in veterinary materials among other habits (Holzapfel and Wood, 2014). *Fructobacillus* species have originally been found in fructose-rich habitats such as flowers, fruit surfaces, fermented fruits, and the guts of insects (Holzapfel and Wood, 2014; Endo et al., 2018). *Lactobacillus* species are usually found in nutrient-rich habitats such as foods, feeds, surface of plants, animals and humans. The ecological role in nature of plant-associated lactobacilli is still poorly understood, as their occurrence is only sporadic they are not considered plant symbionts but rather epiphytic by some researchers (Duar et al., 2017). Duar et al. (2017) classified *Lb. plantarum* and *Lb. rhamnosus* as nomadic lifestyle species while *Lb. brevis* as free-living species. Leuconostocs are associated with different habitats; *Leuc. citreum* has been isolated from clinical sources and from food such as kimchi and wheat sourdoughs; in general, the typical habitats of *Leuc. mesenteroides* subsp. *dextranicum* and *Leuc. mesenteroides* are fermentable plant raw materials, fruit/vegetable mashes (e.g., sauerkraut) and fruit juices; *Leuc. pseudomesenteroides* has been normally isolated from dairy, food, and clinical sources (Holzapfel and Wood, 2014). *Weissella* species inhabit diverse niches such as vegetable-, fish-, and meat-derived fermented foods; *W. cibaria* has been detected in fermented foods of vegetable origin as well as *W. fabalis*, which has been isolated from cocoa bean fermentation; *W. minor* has been found from sludge of milking machine, fermented fruits and vegetables, and fermented dry salami (Fessard and Remize, 2017). *Lactococcus* species have been found on the surface of plants and animals and products of these origins, but the best recognized habitats are raw milk, cheese, and other dairy products (Holzapfel and Wood, 2014).

In the present study, the most abundant species according to the number of isolates were *Leuc. pseudomesenteroides* (155) and *F. tropaeoli* (140), the latter being restricted to samples of custard apple, fig, and khaki. These three fruits possess the highest total sugar content values (12.87, 16.26, and 12.53 g per 100 g of fruit, respectively) from the eight fruits studied here (USDA Food Composition Databases, United States Department of Agriculture, Agricultural Research Service, www.ndb.nal.usda.gov/ndb/). In addition, fig and khaki were the samples from which the majority of LAB were isolated (Table 3). In contrast to these results, the species most frequently reported in these matrices were *W. cibaria*/*confusa* and especially *Lb. plantarum*. Although the latter is considered ubiquitous and is frequently the major part of the lactic population of fruits and vegetables due to its metabolic versatility (Di Cagno et al., 2015), only 3 *Lactobacillus* species were isolated from the samples of our study: *Lb. plantarum* (1 strain, guava), *Lb. brevis* (4 strains, guava; 1 strain, custard apple flower), and *Lb. rhamnosus* (2 strains, fig). *Lb. rhamnosus* is often a dairy-associated species but it has been isolated once from fruit or vegetable by other authors (Ceapa et al., 2015). Similarly, Di Cagno et al. (2010) reported the isolation of *Lb. rossiae* from pineapple although it is a species frequently isolated from sourdough; in another study the meat-associated species *Lb. curvatus* was isolated from pepper by the same authors (Di Cagno et al., 2010). On the other hand, Verón et al. (2017) only isolated strains of *Lb. plantarum* (2) and *F. fructosus* (2) from samples of prickly pear fruit. Askari et al. (2012) reported that *Lactobacillus* and *Leuconostoc* were the most abundant genera in raisins and figs, followed by *Streptococcus* and to a lesser extent *Pediococcus*, whereas in fresh grape juice Bae et al. (2006) found that *Lb. lindneri* was the most frequent species and in a smaller ratio *Lb. kunkeei* and *Ec. durans*. In the fig samples of the present study, *Fructobacillus* was the most abundant genus (4 different species), whereas *Enterococcus, Lactobacillus, Lactococcus*, and *Leuconostoc* were only represented by one strain. Some FLAB have been naturally found in figs (Antunes et al., 2002; Endo et al., 2009). *Lb. plantarum* was also the dominant species in pineapple while *Lb. rossiae* and *W. cibaria* were also present (Di Cagno et al., 2010); Emerenini et al. (2013) identified as *Lb. plantarum* 8 out of 22 LAB from fruits and vegetables while this species was also the most abundant in fruit pulps (Garcia et al. (2016) and fresh fruits (Naeem et al. (2012). Nyanga et al. (2007) found that the lactic microbiota present in samples of mulberries was composed of *Ec. durans, Ec. faecium, Leuc*. *mesenteroides*, and *Leuc*. *pseudomesenteroides*, whereas Chen et al. (2010) found that *W. cibaria* was the dominant species in mulberries from Taiwan together with strains of *Lb*. *plantarum, Lc. lactis* and, similarly to this work, *Leuc*. *pseudomesenteroides*. In the papaya samples, *Enterococcus* was the predominant genus (*Ec. casseliflavus, Ec*. *faecium*, and *Ec*. *hirae*), whereas strains of *Lc. lactis* and *Leuc*. *mesenteroides* were also isolated; other authors isolated strains of *W. cibaria, Lb. plantarum, Lb. pentosus, Leuc. holzapfelii*, and *Leuc. mesenteroides*/*pseudomesenteroides* (Fessard et al., 2016). Custard apple fruits showed a broad LAB species diversity, such as *Ec. hirae, Ec. mundtii, F. durionis, F. tropaeoli, Leuc. pseudomesenteroides*, and *W. cibaria*, although a low number of isolates were obtained. The only authors who reported LAB isolation from custard apple were Trias Mansilla et al. (2008), who found one strain of *Leuc. mesenteroides*. Regarding the presence of LAB on flowers, *Ec. casseliflavus* was isolated from the 3 types of flowers analyzed in our study, whereas *Lc. lactis* was present on flowers of passion fruit and medlar. On medlar flowers, as well as on the fruits, *Leuc. pseudomesenteroides* was present, whereas on the passion fruit flowers a *Leuc. mesenteroides* strain was found and *W. cibaria* was the most abundant species. Other *Lactobacillus* species, such as *Lb. ozensis* (Kawasaki et al., 2011a), *Lb*. *florum* (Endo et al., 2010), *Lb. floricola* (Kawasaki et al., 2011b), *Lb. sakei* and *Lb. kunkeei* (Linjordet, 2016) in addition to *Ec. faecium* (Di Cagno et al., 2011a) and *Enterococcus* spp. (Linjordet, 2016) were isolated from wild flowers. Linjordet (2016) conducted a detailed study, during which *W. viridescens* and *W. ceti, Lc. lactis, Lc. garvieae*, and *F. fructosus* were isolated. Some of these LAB species, such as *F. fructosus, Lb. kunkeei*, and *Ec. faecium*, has also been isolated from the pollinator *Apis mellifera* L. (Carina Audisio et al., 2011; Pachla et al., 2018). The lactic microbiota of guava, passion fruit, medlar, khaki, and flowers of passion fruit, custard apple, and medlar was reported for the first time during the present study. No new LAB species could be isolated from the samples studied.

The carbohydrates present in the isolation media as well as the isolation methods employed strongly influence the success for the isolation of LAB from specific niches. Direct isolation is generally used if cells are present in high numbers, as in feces and fermented foods; however, if microbial cells are present in low numbers and specific species are sought, culture enrichment before bacterial isolation should be applied. This methodology presents the disadvantage that only some species, showing fast cell growth, will grow at the cost of others, eliminating competition from those with slower growth. One of the main factors responsible for this selection is the growth substrate, since the use of specific carbon sources is generally different at species level (Endo et al., 2011a). In this work, culture enrichment using fructose as a carbon source for the isolation of FLAB was conducted. This approach enabled not only the isolation of FLAB but also other LAB species which were present in very low numbers. Several authors isolated LAB from flowers and fruits using this methodology (Antunes et al., 2002; Bae et al., 2006; Endo et al., 2009, 2010, 2011a; Naeem et al., 2012).

In general, glucose is the most easily metabolizable substrate for the majority of microorganisms, including LAB, and therefore the most used carbohydrate for bacterial isolation and culturing (Antunes et al., 2002). However, several studies have suggested that some species have evolved by adapting to their niches to survive and preferring to metabolize other specific carbohydrates; this may be the case for FLAB that, when inhabiting fructose-rich niches such as fruits, may have lost their ability to mainly metabolize glucose during adaptation and hence preferring fructose as a carbon source to grow (Endo et al., 2011a; Endo, 2012; Filannino et al., 2019). In our work, the use of fructose allowed isolating the fructophilic species *F. fructosus* and *F. pseudoficulneus*, and to increase the number of fructophilic isolates of *F. durionis* and *F. tropaeoli*. Endo et al. (2009) described the isolation protocol for FLAB, by which they could isolate *F. pseudoficulneus* from banana and fig samples and *Lb. kunkeei* and *F. fructosus* from different flowers.

It has been claimed that the microbial population present on plants and their parts, including flowers and fruits, may be subjected to nutritional fluctuations, and physicochemical and environmental conditions, as well as to dispersal events (Samuni-Blank et al., 2014), reasons by which each fruit, flower or plant might carry a particular microbiota in a specific geographical region and on a specific time-point. Plant-associated habitats (roots, leaves, flowers, fruits or decaying tissues) differ in their local availability of nutrients and physicochemical conditions, conditioning the range of potential microbiota. For instance, floral nectar has been regarded merely as a sweet aqueous secretion offered by flowering plants to attract pollinators. Nevertheless, pollinators act not only as pollen vectors, but at the same time they can transport microorganisms from flower to flower (Alvarez-Perez et al., 2012). Also, it has been demonstrated that nectar microbial community may vary among different plant species (Fridman et al., 2012). All this background, might explain the variations regarding LAB diversity present on flowers and fruits reported in the literature so far.

Recently, there has been particular interest in the use of autochthonous fruit LAB as starter cultures to be used in the manufacture of differentiated and/or functional fruit-based foods (Trias et al., 2008; Di Cagno et al., 2011a,b, 2015, 2016; Mousavi et al., 2011; Filannino et al., 2013). Fruits and flowers are fructose-rich plant parts that heterofermentative LAB inhabit; many of these bacteria being able to reduce this sugar and to produce mannitol (Filannino et al., 2018). From 24 positive mannitol-producing strains, 6 strains of the genera *Leuconostoc* and *Fructobacillus* synthesized mannitol in concentrations higher than 5 g/L from 10 g/L of fructose present in the culture medium. According to the literature, several strains of *Lactobacillus, Leuconostoc, Fructobacillus*, and *Oenococcus* are capable of producing mannitol from fructose (Saha and Racine, 2011). Although to date *Lb. intermedius* NRRL B-3693 is the LAB strain with the highest capacity to produce mannitol reported so far (Saha and Nakamura, 2003; Saha, 2006a,b,c; Saha and Racine, 2010), strains of *Leuconostoc* and *Fructobacillus* have been described as very good mannitol producers (von Weymarn et al., 2002; Fontes et al., 2009; Carvalheiro et al., 2011; Ruiz Rodríguez et al., 2017b). Likewise, all described species of the genus *Fructobacillus*, normally isolated from fructose-rich niches such as flowers, fruits and insect intestines, can convert fructose into mannitol as a result of their peculiar fructophilic metabolism (Endo et al., 2009, 2014; Endo, 2012). Filannino et al. (2016) reported that all FLAB strains isolated from bee intestines, including 5 strains of *F. fructosus*, produced mannitol from fructose. Other plant-associated species such as *Lb. florum* were capable of producing mannitol in culture media containing glucose and fructose (Tyler et al., 2016), and as observed for *F. tropaeoli* F-H3-450 and *F. fructosus* F-H2-401 during the present study.

Diacetyl is undoubtedly another industrially interesting compound that contributes to the flavor of many fermented foods and can be naturally synthesized by LAB. The ability to form diacetyl was present in all genera, except for *Weissella*; strains of *Lb. rhamnosus* showing the highest production. The most important diacetyl-producing LAB species are *Lc. lactis, Lactobacillus* spp., *S. thermophilus*, and *Leuc. mesenteroides*, besides the subspecies *Lc. lactis* biovar. *diacetylactis* (Ruiz Rodríguez et al., 2017a). Filannino et al. (2014) studied the behavior of strains of *Lb. plantarum* isolated from fermented fruits and vegetables and found that diacetyl was the ketone present in the highest concentration.

Some LAB possess esterase activity (Liu et al., 2014), capable of hydrolyzing and producing esters, which play a fundamental role in the flavor of fermented foods. In the present work, the EA_h_ was strain-specific; in addition, substrate specificity was different among different LAB genera and species. Although not many studies on LAB strains isolated from fruit sources exist, this observation coincides with that reported for strains using other matrices (Oliszewski et al., 2007; Pérez-Martín et al., 2013). The EA_h_ on α-NA-C2, α-NA-C3, α-NA-C4, and α-NA-C8 derivatives was distributed among the LAB strains studied; however, a tendency of the strains to be more active against short-chain substrates such as α-NA-C2, α-NA-C3, and α-NA-C4 was found. Similarly, Oliszewski et al. (2007) reported that all strains of indigenous goat milk and cheese LAB showed EA_h_ on α-NA-C2 to α-NA-C6. Matthews et al. (2007) also found that 9 strains of *Oenococcus, Lactobacillus*, and *Pediococcus* species, isolated from commercial starter cultures for vinification and from olive products, showed greater activity on short-chain esters (C2-C8) compared to long ones (C10-C18). Likewise, Taboada et al. (2014) found that 22 LAB strains from goat dairy products showed activity on α-NA-C2, α-NA-C3, α-NA-C4 and α-NA-C8. The preferential or exclusive hydrolysis of α-NA esters derived from C2-C6 fatty acids by LAB was detected by other authors too (Gobbetti et al., 1996; Katz et al., 2002). On the contrary, Pérez-Martín et al. (2013) observed that most of the 243 wine strains of *Oenococcus, Lactobacillus, Pediococcus*, and *Enterococcus* species from different grape varieties could more easily hydrolyse esters of 8 and more carbon atoms. The EA_h_ values determined in the present work were varied, the *Lc. lactis* and *Lb. rhamnosus* strains showing the maximum activity values, whereas the lowest corresponding to strains of *Lactobacillus* and *Enterococcus* species. Oliszewski et al. (2007) reported that enterococci showed the highest activities on α-NA-C4 and α-NA-C6, whereas *Lb. rhamnosus* ETC14 showed the highest specific EA_h_ on α-NA butyrate and caproate. Taboada et al. (2014) also reported that a *Lb. rhamnosus* strain showed the highest activity on α-NA acetate. Similarly, the strain *Lb. rhamnosus* H3F-210 of our study showed the highest activity values on α-NA-C3 and α-NA-C4. Nardi et al. (2002) found that the esterase activity EstA was responsible for most of the ester-producing activity in *Lc. lactis*. Most of the strains of LAB species present in wine, namely *Oenococcus, Pediococcus* and *Lactobacillus*, possess esterase activity (Sumby et al., 2010). Further, several studies highlight the biosynthesis of fruity esters by *O. oeni* strains (Costello et al., 2013; Sumby et al., 2013).

As mentioned elsewhere, in addition to hydrolysis, esterases also have the ability to synthesize esters by esterification of fatty acids and ethanol. These ethyl esters, even in very low amounts, play an important role in the development of the fruity organoleptic characteristics of some foods (Taboada et al., 2014). In this work, the ability to produce ethyl esters through esterification by 8 selected LAB strains with EA_h_ was evaluated. REA values were variable and dependent on both the strain and substrate used, in coincidence with the findings of Abeijón Mukdsi et al. (2009) and Costello et al. (2013), working with LAB strains from milk and goat and sheep cheese origin and wines, respectively. When studying particularly the biosynthesis of ethyl butanoate by dairy LAB, (Liu et al., 1998) found that this ability was variable and species- and strain-dependent. All the strains studied in the present work could synthesize at least one ethyl ester from the two substrates assayed as well as the strains studied by (Costello et al., 2013). The fruit- and flower-origin strains of the present study produced ethyl acetate, ethyl propionate, and ethyl butanoate from butanoic acid and hexanoic acid, unlike the vinification LAB strains that produced mainly ethyl butanoate, ethyl hexanoate, and ethyl octanoate from the corresponding fatty acid precursors (Costello et al., 2013), and of other dairy-origin strains that produced mainly ethyl butanoate and ethyl hexanoate (Abeijón Mukdsi et al., 2009). All the strains tested produced ethyl acetate, although to a different extent, in contrast to the observations of Abeijón Mukdsi et al. (2009), who did not detect the biosynthesis of this ethyl ester by LAB. In the present work, the studied strains produced 3 types of ethyl esters when butanoic acid was used as substrate, unlike when using hexanoic acid, which led to the biosynthesis of ethyl acetate (2 strains) and ethyl propionate (2 strains). In contrast, Abeijón Mukdsi et al. (2009) found that some strains could synthesize mainly EtC6 from butanoic acid, whereas Costello et al. (2013), in agreement to the present findings, did not detect biosynthesis of EtC4 from the corresponding fatty acid. Regarding the biosynthesis of ethyl butanoate, strains of *Lb. rhamnosus, Lc. lactis*, and *Lb. brevis* showed the highest biosynthesis activity, whereas strains of *Leuc. mesenteroides* and *Leuc. pseudomesenteroides* showed the lowest capacity. Also, Liu et al. (1998) observed that strains of *Lc. lactis* showed moderate ethyl butanoate biosynthesis activity and strains of *Lb. rhamnosus* and *Lb. paracasei* subsp. *paracasei* showed greater potential than those of lactococci, whereas the capacity of *Leuconostoc* strains was variable.

As mentioned above, all strains studied produced ethyl acetate from butanoic acid, which is not a direct precursor of this ester. The mechanism involved in this phenomenon is still unknown. Some authors (Liu et al., 2003; Abeijón Mukdsi et al., 2009) suggested that other mechanisms of ester biosynthesis in addition to esterification could be involved; Liu et al. (2004) hypothesized that ethyl esters could be synthesized non-enzymatically.

Noticeably, when comparing the EA_h_ and REA activities of the strains of this study, their ability to hydrolyze esters of a certain length was not always correlated with the capacity to produce ethyl esters of the same length (Figure 4); these results may be explained by the presence of more than one esterase enzyme with different specificities (Oliszewski et al., 2007).

Native cultures are preferred to allochthonous starters for food fermentation since indigenous strains display shorter latency phases and better acidification capacity. For the selection of autochthonous strains as starter cultures for fruit and vegetable fermentations, the bacterial capacity to lower the matrix pH to values below 4.5 should be considered essential to achieve the inhibition of unwanted microorganisms from the early stages of fermentation (Di Cagno et al., 2013). Since rapid growth and acidification rates are conventional criteria for the selection of starter cultures, these parameters were studied. In general, acidification kinetics and growth parameters were variable among the LAB strains examined, in coincidence with findings of LAB from plant matrices (Filannino et al., 2014; Fessard et al., 2016, 2017). Although the absolute values of the parameters studied were strain-dependent, a slight tendency of lactococci and fructobacilli to grow and acidify more rapidly than the other bacteria was noticed, the lowest values being observed for *Weissella* and *Lactobacillus* strains. Fessard et al. (2016) studied 10 LAB strains isolated from papaya, tomato, and cabbage, belonging to *Leuconostoc, Weissella* and *Lactobacillus*, and found V_max_ values of 0.10- 0.15 U pH/h when growing the strains in MRS. These values were lower than those found in the present work and much lower than for other LAB (0.2-1.2 U pH/h) (Latrille et al., 1992; Xanthopoulos et al., 2001; Cachon et al., 2002). The same researchers found that strains of *W. cibaria* showed the highest acidifying potential. More recently, Fessard et al. (2017) observed that half of 28 strains of *Leuconostoc, Lactococcus, Lactobacillus*, and *Fructobacillus* examined exhibited V_max_ values in the range of 0.155–0.22 U pH/h. Analyzing some particular cases in our study, the strain of *Lb. plantarum* showed values of V_max_ = 0.21 U pH/h, similar to those found by Filannino et al. (2014) for *Lb. plantarum* strains isolated from cherry and pineapple in MRS (0.23- 0.21 U pH/h, respectively). Although acidification kinetics has been widely used as a tool to monitor fermentation performance, this parameter it is not frequently used in vegetable or fruit fermentations and the LAB starters related to them (Fessard et al., 2016). Different microbial acidifying capacities are needed depending on the type of fermented product; on one side, rapid acidification (i.e., *L. plantarum*) till pH ≤ 4.5 is desired in fruit juice fermentation (Di Cagno et al., 2010) while on the other side, for a few vegetables mild acidification is preferred (Lee et al., 2011) as in the case of kimchi, where over souring is one of the most serious defect. Then, autochthonous strains of *Lb. sakei* are selected because of their mild acid-producing properties (Lee et al., 2011). Thus, the diversity on the growth and acidification parameters shows the potential of the strains of our study to be used in starter culture formulations for different fermentation processes.

In nature, several microorganisms are capable of synthesizing pectinases, a complex set of hydrolytic enzymes that cleave pectic substances that constitute a large part of the vegetable raw materials (Sakellaris et al., 1989; Karam and Belarbi, 1995; Pedrolli et al., 2009; Prathyusha and Suneetha, 2011). Although this microbial enzymatic activity may cause unwanted softening of fermented vegetables, as in pickles (Buckenhüskes, 1993), this pectinolytic activity would be desirable in starters in the fruit juice industry, since it would help to control the viscosity of these products (Di Cagno et al., 2013). Currently, little information about the pectinolytic activity of LAB is available although several pectinase and pectinase-like coding genes have been recently annotated (http://www.ncbi.nlm.nih.gov/GenBank/). In our work, 43 strains representative of the 6 genera assayed capable of hydrolyzing citrus pectin were found. Sakellaris et al. (1988, 1989) described the extracellular polygalacturonase (PG) activity of a *Lb. plantarum* strain and purified and characterized this enzyme. Karam and Belarbi (1995) studied the presence of pectinolytic activity in 80 LAB strains isolated from milk in Algeria, of which only 4 strains (2 *Lb. casei*, 1 *Lb. plantarum*, and 1 *Lc. lactis*) produced PG and/or pectin esterase. Vidhyasagar et al. (2013) studied pectin degradation by LAB isolated from fermented foods and found strains of *P. pentosaceus, Leuc*. *lactis* and *Lb. plantarum* subsp. *argentoratensis* able to hydrolyze pectin. Chatterjee et al. (2016) found that the presence of pectin significantly improved bacterial growth and titratable acidity in LAB and *Bifidobacterium* cultures, concluding that it could be used as a potential prebiotic. The pectinolytic activity of strains of *Enterococcus* and *Fructobacillus* species was qualitatively revealed for the first time in the present work. Further studies are needed to characterize these enzymes.

Considering that human tissues and biological fluids do not possess esterases capable of hydrolyzing esters of phenolic acids (for example, chlorogenic acid), bacterial cinnamoyl esterases present in starter cultures could enrich plant matrices in free phenolic acids with high bioavailability for man (Filannino et al., 2018). For this reason, the presence of cinnamoyl esterases in LAB from flowers and fruits was evaluated. Only 14% of the examined strains showed weak cinnamoyl esterase activity compared to the control. The species capable of hydrolyzing ethyl ferulate were *Lc. lactis, W. minor, Lb. plantarum, Ec. casseliflavus, Leuc. mesenteroides*, and *Leuc. pseudomesenteroides*, being the strain *Lc. lactis* F-Cq1-484-2 the most remarkable whose halo, although larger and more defined than the others, was less evident than that of the positive control. Many of the strains with enzymatic activity on phenolic compounds were isolated from raw materials or fermented foods with a high content of these compounds or from human samples (Sánchez-Maldonado et al., 2011; Di Cagno et al., 2015). Abeijón Mukdsi (2009) isolated the strain *Lb. fermentum* CRL 1446 with high cinnamoyl ester activity, mainly on methyl ferulate, from an Argentinean goat cheese, whereas Xu et al. (2017) isolated 4 strains of *Lactobacillus* with feruloyl esterase activity from ensiled corn stover. Esteban-Torres et al. (2013) studied the ability to hydrolyze hydroxycinnamic esters by the human saliva strain *Lb. plantarum* WCFS1 and found that it could partially hydrolyze methyl ferulate and methyl p-coumarate. Later, the same authors (Esteban-Torres et al., 2015) studied the cinnamoyl ester activity of 28 *Lb. plantarum* strains; only 7 could hydrolyze methyl ferulate or methyl caffeate. To date, studies on cinnamoyl esterase activity of LAB strains isolated from flowers and fruits remain scarce. Interestingly, data on *Lc. lactis, W. minor, Ec. casseliflavus, Leuc. mesenteroids*, and *Leuc. pseudomesenteroides* strains with esterase activity on hydroxycinnamic acids was reported in the present study for the first time.

Histamine is recognized as the causative agent of scombroid poisoning, whereas tyramine consumption has been linked to food-induced migraines and hypertensive crisis in patients who consume monoamine oxidase inhibitor drugs. In turn, putrescine and cadaverine can potentiate the toxicity of the previous amines and, in addition, be precursors of carcinogenic nitrosamines. Apart from these toxicological aspects, the appearance of relatively high levels of certain BA indicates deterioration and/or defective food elaboration (Bover-Cid and Holzapfel, 1999). From all strains evaluated, only three (*Lb. lactis, Leuc. mesenteroides* and *Leuc pseudomesenteroides*) produced only tyramine, indicating that the formation of BA was not widely spread among the fruit- and flower-origin LAB assayed. Similarly, tyramine was the main BA formed by *Enterococcus, Carnobacterium*, and some *Lactobacillus* strains in the studies conducted by Bover-Cid and Holzapfel (1999) and by *Leuconostoc* strains in the work of Moreno-Arribas et al. (2003). To date, very few strains of *Lb. curvatus, Lb. buchneri*, and *Lb. brevis* as producers of putrescine and cadaverine were detected (Bover-Cid and Holzapfel, 1999; Moreno-Arribas et al., 2003) and only significant levels of histamine biosynthesis were reported in strains of *O. oeni* (Landete et al., 2005). On the other hand, Tomita et al. (2017) found that heterofermentative LAB species were characterized as producers of high levels of tyramine.

## Conclusion

The results obtained during the present study supported the hypothesis that LAB strains isolated from fruits and flowers from Northern Argentina could be exploited from a biotechnological point of view. Strains capable of producing mannitol, organic acids, and aroma compounds were found; in addition, strains harboring cinnamoyl esterase, pectinase, and esterase activities, interesting properties to be used in fruit food matrices, were detected. Differences between the results obtained for the fruit- and flower-origin LAB strains of the present study and those available in the literature could be explained by the diversity of substrates, fermentation protocols, and analyses used; but more importantly, they could be inherent to the microbial diversity existing in wild niches belonging to different regions of the world. In this sense, this work provided a deeper insight into the lactic microbiota present on tropical fruits and flowers. In addition, the LAB strains isolated harbored interesting functional properties to be used in starter culture formulations for fruit-based fermented food products.

## Author Contributions

LRR carried out the majority of the experiments and wrote the manuscript. FM and JB carried out some of the experiments. RM directed the esterases experiments. LDV and EH corrected the manuscript. FeM directed the work and corrected the manuscript.

### Conflict of Interest Statement

The authors declare that the research was conducted in the absence of any commercial or financial relationships that could be construed as a potential conflict of interest.
