# Supplementary material for: Diversity and Functional Properties of Lactic Acid Bacteria Isolated From Wild Fruits and Flowers Present in Northern Argentina
Source: Front Microbiol. 2019 May 21;10:1091. doi: 10.3389/fmicb.2019.01091 (PMC6536596; doi:10.3389/fmicb.2019.01091)
Supplement: Supplementary file 1 [file Table_1.DOCX]

Table S1. Main weather features of fruit and flower sampling months.

| **Sampling month** | | **T** | **TM** | **Tm** | **H** | **PP** | **V** | **RA** |
| --- | --- | --- | --- | --- | --- | --- | --- | --- |
| **2013** | April | 20.3 | 27.9 | 15.2 | 72.4 | 198.11 | 9.2 | 5 |
|  | June | 14.6 | 22.2 | 9.1 | 69.4 | 21.07 | 10.7 | 6 |
|  | July | 12.9 | 21.3 | 6.9 | 61.8 | 1.27 | 10.4 | 3 |
|  | August | 14.7 | 24.6 | 7.1 | 41.8 | 0 | 12 | 1 |
|  | September | 18.3 | 27.9 | 11.7 | 45.4 | 61.72 | 12.5 | 4 |
|  | October | 23.4 | 32.1 | 16.2 | 51.4 | 11.68 | 12 | 7 |
| **Annual** |  | 20.6 | 28.6 | 14.6 | 61.2 | 1076.18 | 10.9 | 83 |
| **2014** | January | 26.4 | 34.2 | 20.4 | 67.2 | 192.8 | 11.8 | 12 |
|  | April | 19.9 | 25.7 | 16.2 | 80.6 | 17.78 | 8.9 | 15 |
| **Annual** |  | 20.4 | 27.6 | 15.1 | 69.5 | -* | 10.8 | 125 |

**T**: average temperature (°C). **TM**: maximum temperature (°C). **Tm**: minimum temperature (°C). **H**: average relative humidity (%). **PP**: total precipitation of rain or melted snow (mm). **V**: average wind speed (Km/h). **RA**: indicates if there was rain or drizzle (on the average per month, total days rained).

*No data available for the period.
