# Supplementary figures and images for: Diversity and Functional Properties of Lactic Acid Bacteria Isolated From Wild Fruits and Flowers Present in Northern Argentina
Source: Front Microbiol. 2019 May 21;10:1091. doi: 10.3389/fmicb.2019.01091 (PMC6536596; doi:10.3389/fmicb.2019.01091)

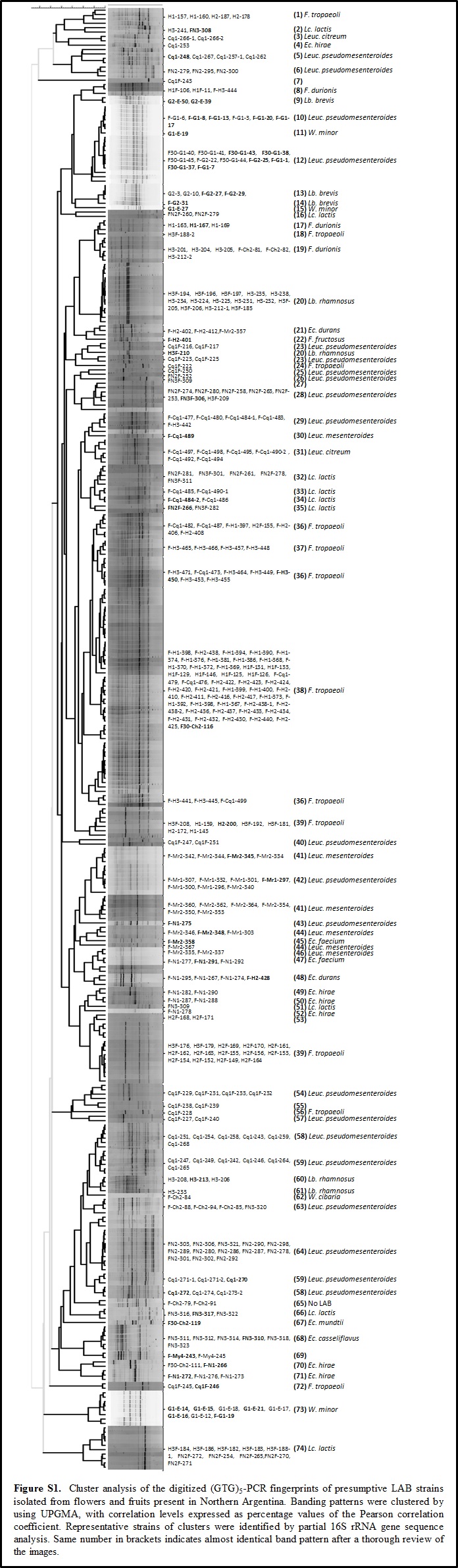

Supplement: Supplementary file 2 [file Image_1.JPEG]

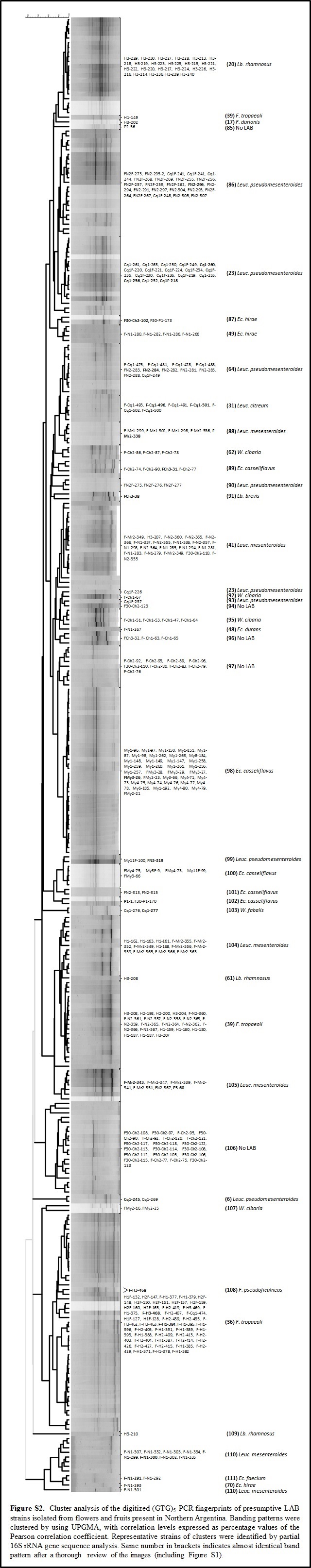

Supplement: Supplementary file 3 [file Image_2.JPEG]
